# Supplementary material for: Epithelial SIRT6 governs IL-17A pathogenicity and drives allergic airway inflammation and remodeling
Source: Nat Commun. 2023 Dec 22;14:8525. doi: 10.1038/s41467-023-44179-x (PMC10746710; doi:10.1038/s41467-023-44179-x)
Supplement: Supplementary file 1 — Supplementary Information [file 41467_2023_44179_MOESM1_ESM.pdf]

Supplemental figures and tables

Supplementary Fig. 1

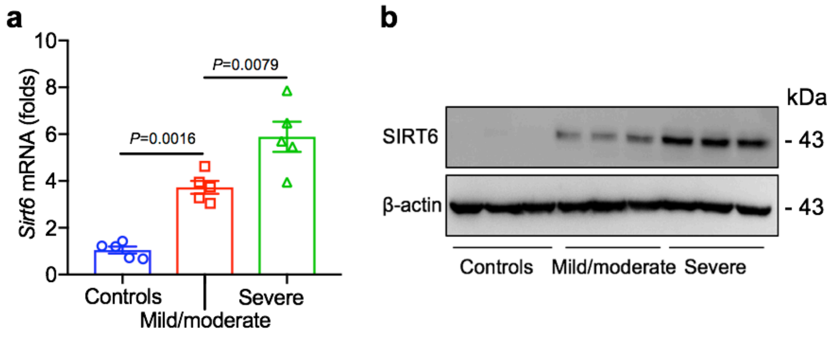

Supplementary Fig. 1 SIRT6 expression in human bronchial biopsy samples. SIRT6 expression in the bronchial biopsy samples from control subjects (n = 5), mild/moderate asthma (n = 5), and severe asthma (n = 5) was assessed using qRT-PCR (a) and Western blot (b). Data are shown as means ± SEM and three or more independent experiments were performed. Significance was calculated by one-way ANOVA followed by Tukey's post-hoc test for (a).

Supplementary Fig. 2

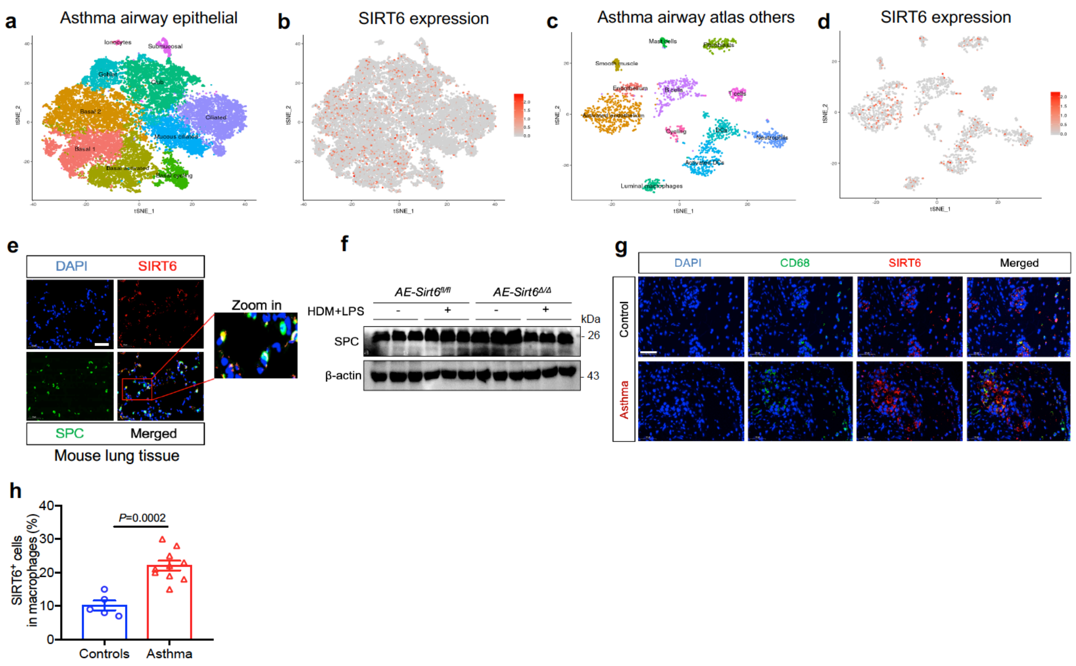

Supplementary Fig. 2 SIRT6 expression in different types of cells. a-d Using published single-cell RNA-seq (scRNA-seq) data [Nat Med. 2019; 25:1153-1163], after quality control and first dimensionality reduction clustering, epithelial cells and other types of cells were extracted for secondary dimensionality reduction clustering. The analysis of database revealed that airway epithelial cells and other cells such as macrophages, different T cell populations have an expression of SIRT6. **e** SIRT6 (red) expression in alveolar epithelial cells (SPC, green) of the *AE-Sirt6*$^{fl/fl}$ mice using IF staining. Scale bars, 100 μm. **f** Western bolt for SIRT6 expression in the lung tissue from *AE-Sirt6*$^{fl/fl}$ and *AE-Sirt6*$^{\Delta/\Delta}$ mice treated with or without HDM/LPS. **g-h** SIRT6 (red) expression in macrophages (CD68, green) of bronchial biopsy samples from control subjects (n = 5) and asthmatic patients (n = 10) was assessed using IF staining. Scale bars, 100 μm. Data are shown as means ± SEM and three or more independent experiments were performed. Significance was calculated by Two-tailed unpaired Student's t test for (h).

Supplementary Fig. 3

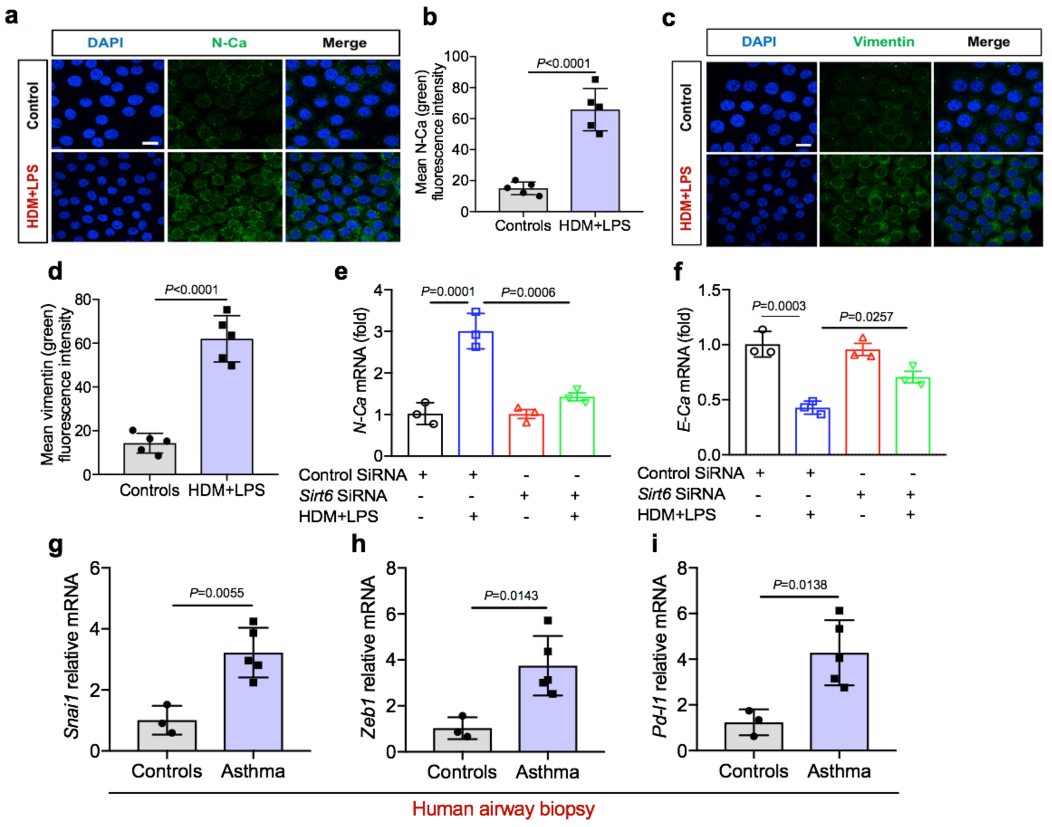

Supplementary Fig. 3 Allergen exposure induces EMT in asthmatic patients. a-b Representative IF staining of N-Ca in HBE cells stimulated with allergen. Scale bars, 20 µm. Quantification of IF staining was done by using Image J software. **c-d** Representative IF staining of vimentin in HBE cells stimulated with HDM/LPS. Scale bars, 20 µm. Quantification of IF staining was done by using Image J software. **e-f** HBE cells were transfected with SIRT6 siRNA for 24h and then treated with allergen (HDM/LPS) for another 48 h. *N-ca* and *E-ca* were analyzed using qRT-PCR. **g-i** qRT-PCR for bronchial mucosal biopsy specimens from control subjects (n = 5) or patients with asthma (n = 10) for the EMT regulators *Snai1*, *Zeb1*, and *Pd-l1* mRNA expression. Data are shown as means ± SEM and three or more independent experiments were performed. Significance was calculated by Two-tailed unpaired Student's t test for (b, d, g-i) and one-way ANOVA followed by Tukey's post-hoc test for (e, f).

Supplementary Fig. 4

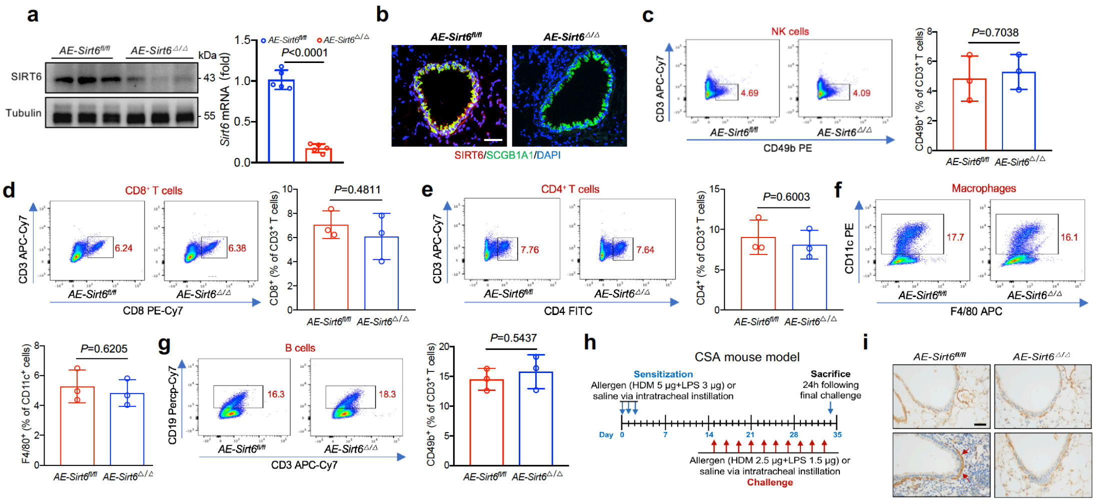

Supplementary Fig. 4 Establishment of airway epithelial cell-specific *Sirt6* knockout mice. a Western blot analysis of SIRT6 abundance in airway epithelial cell-specific *Sirt6* knockout mice (*AE-Sirt6*$^{\Delta/\Delta}$) mice compared with control (*AE-Sirt6*$^{fl/fl}$). **b** Representative micrographs of IF staining for SIRT6 (red), nuclei (DAPI, blue), and airway epithelial cells (SCGB1A1, green) in lung sections of *AE-Sirt6*$^{fl/fl}$ and *AE-Sirt6*$^{\Delta/\Delta}$ mice. Scale bars, 50 μm. **c-g** Representative flow plots are shown for NK cell, CD8^{+} T cell, CD4^{+} T cell, Macrophage, and B cell subsets isolated from the lung tissue of *AE-Sirt6*$^{fl/fl}$ and *AE-Sirt6*$^{\Delta/\Delta}$ mice. Flow cytometry was performed with Fortessa (BD Biosciences). Quantification was analyzed with FlowJo (TreeStar). **h** Schematic illustrating a chronic severe asthma (CSA) model. **i** Representative α-SMA staining of lung sections from *AE-Sirt6*$^{fl/fl}$ and *AE-Sirt6*$^{\Delta/\Delta}$ mice treated with HDM/LPS. Scale bars, 50 μm. Data are shown as means ± SEM and three or more independent experiments were performed. Significance was calculated by Two-tailed unpaired Student's t test for (a, c-g).

Supplementary Fig. 5

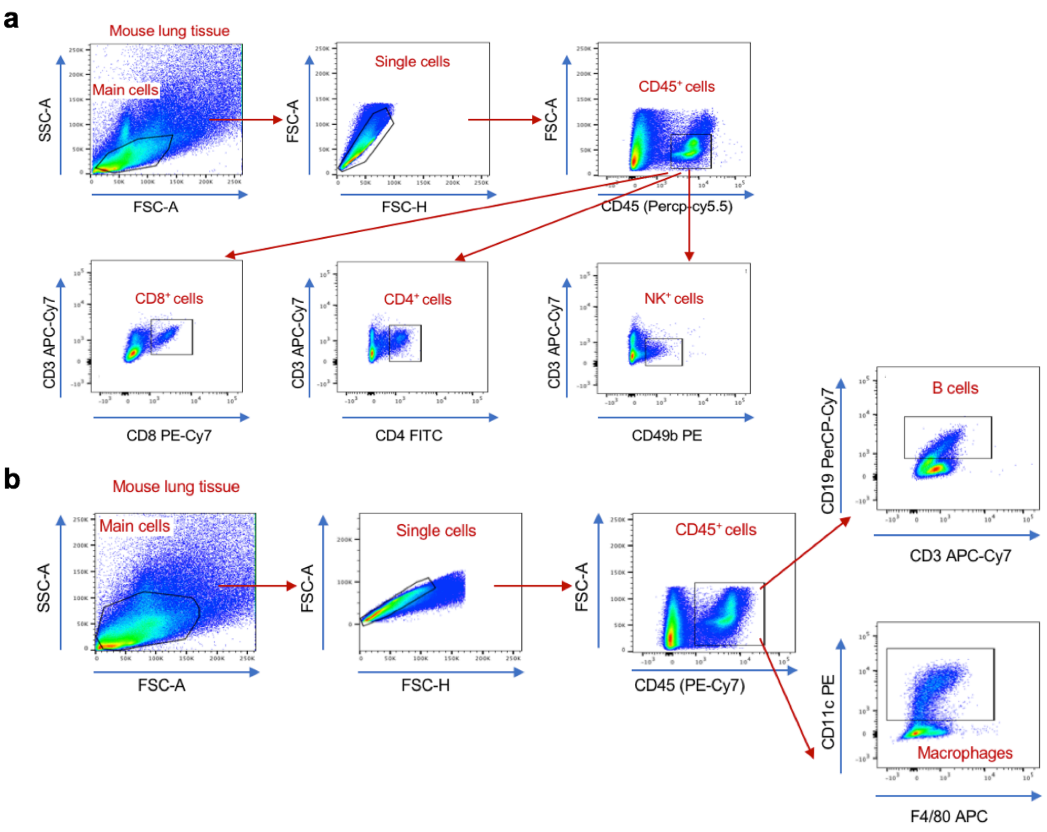

Supplementary Fig. 5 FACS gating strategies for identifying indicated cell populations. **a-b** Mouse lung single-cell suspensions were harvested and stained with fixable viability stain to exclude dead cells. Gating strategies and representative flow cytometry plots to identify the indicated immune cell populations.

Supplementary Fig. 6

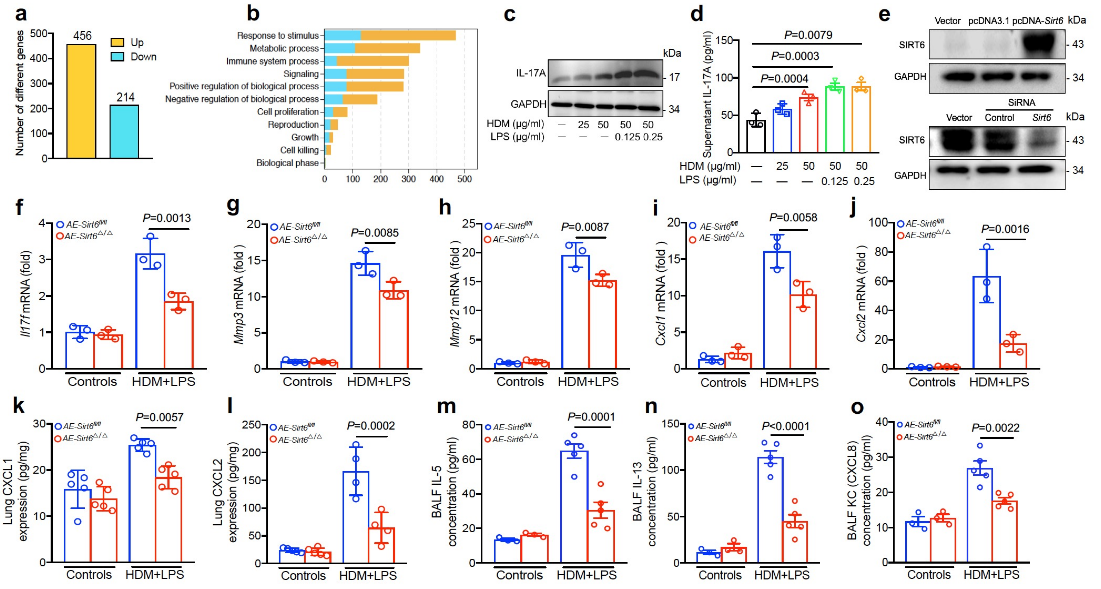

Supplementary Fig. 6 SIRT6 deficiency decreases the expression of MMPs and chemokines. a RNA-seq analysis of RNA isolated from control and asthmatic mice. The differential up- and down-regulation genes were analyzed. **b** Gene Ontology (GO) biological processes enrichment analysis of the significantly differentially expressed genes (DEGs). **c-d** HBE cells were treated with different dose of HDM/LPS for 24h. The expression of IL-17A was studied by using Western blot and ELISA analysis. **e** HBE cells were transfected with *Sirt6* small interfering RNA (siRNA) or *Sirt6* plasmid for 24h. The expression of SIRT6 was studied by using Western blot analysis. **f-l** Inflammatory cytokines and MMPs in the lung homogenate of *AE-Sirt6*$^{fl/fl}$ and *AE-Sirt6*$^{\Delta/\Delta}$ mice were assessed by using qRT-PCR and/or ELISA analysis. **m-o** Inflammatory cytokines in the BALF of *AE-Sirt6*$^{fl/fl}$ and *AE-Sirt6*$^{\Delta/\Delta}$ mice were assessed by using ELISA analysis. Data are shown as means ± SEM and three or more independent experiments were performed. Significance was calculated by one-way ANOVA followed by Tukey's post-hoc test for (d, f-o).

Supplementary Fig. 7

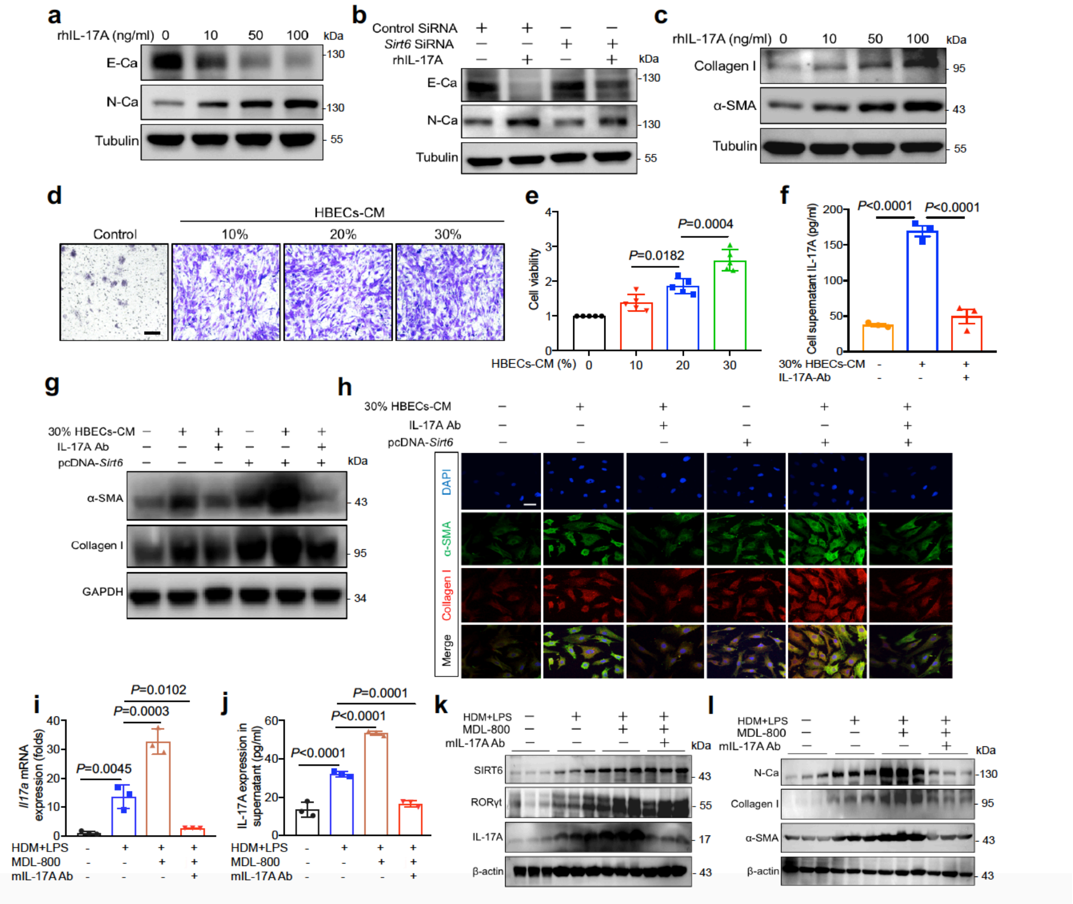

Supplementary Fig. 7 SIRT6 deficiency attenuates airway remodeling via IL-17A-dependent mechanism. a HBE cells cultured with different dose of recombinant human (rh) IL-17A for 24h. EMT makers N-Ca and E-Ca were assessed by Western blot. **b** HBE cells were transfected with *Sirt6* siRNA for 24h and then treated with HDM/LPS and/or rhIL-17A for another 24h. N-Ca and E-Ca were determined by Western blot analysis. **c** Human lung fibroblast Cells (MRC5) cultured with different doses of rhIL-17A for 24h. Myofibroblast markers Collagen I and α-SMA were assessed by Western blot. **d** The culture supernatants derived from the HDM/LPS-treated HBE cells were used to stimulate lung fibroblasts (Scale bars, 50 μm). **e** Cell viability was assessed using CCK8 analysis. **f** IL-17A was determined using an ELISA assay in the culture supernatants derived from the HDM/LPS-treated HBE cells. **g-h** Mouse primary lung fibroblasts were pretreated with *Sirt6* siRNA or *Sirt6* plasmid for 24h and were then treated with HDM/LPS fibroblast-derived supernatants after neutralization of IL-17A (IL-17A Ab) for 48h. The myofibroblast markers α-SMA and

Collagen I were analyzed using Western blot and IF. Scale bars, 50 μm. **i-l** As described in Methods, WT mice were divided into four groups: (i) Control, (ii) HDM + LPS, (iii) HDM + LPS + activator (MDL-800), and (iv) HDM + LPS + MDL-800 + IL-17A neutralization (IL-17A Ab). The expression of IL-17A, SIRT6, RORγt, and the airway remodeling relative markers was measured. Data are shown as means ± SEM and three or more independent experiments were performed. Significance was calculated by one-way ANOVA followed by Tukey's post-hoc test for (e, f, i, j).

Supplementary Fig. 8

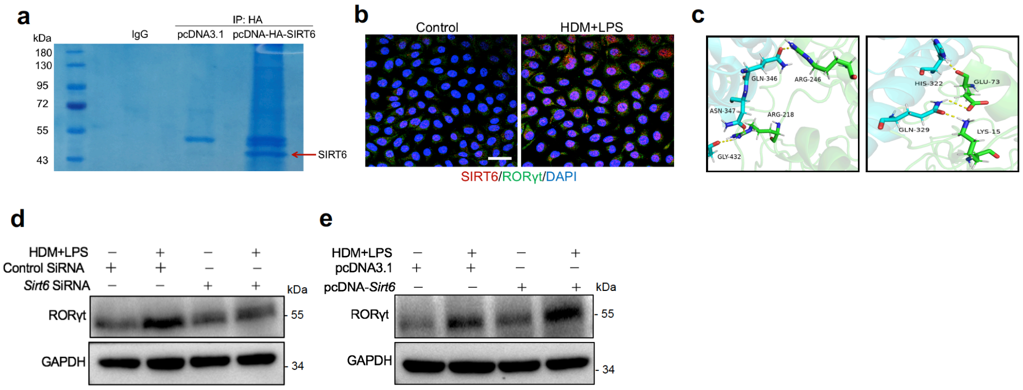

Supplementary Fig. 8 SIRT6 directly interactes with RORγt. a SDS-PAGE of purified proteins immunoprecipitated by anti-SIRT6 antibody. **b** Confocal microscopy of the location of endogenous SIRT6 (Red) and RORγt (Green) in HBE cells treated with HDM/LPS for 24h. DAPI, DNA binding dye. Scale bars, 20 μm. **c** Residues ARG-246 and ARG-218 of SIRT6 form hydrogen-bonding interactions with residues GLN-346, GLY-432, and ASN-347 of RORγt; Similarly, residues GLN-329 and HIS-322 of RORγt interact with residues LYS-15 and GLU-73 of SIRT6 form hydrogen bonding interactions. The two proteins rely more on hydrogen bond interaction under the acetylation modification site to maintain the binding. **d, e** HBE cells were pretreated with *Sirt6* siRNA or *Sirt6* plasmid for 24h and then treated with HDM/LPS for another 24h. RORγt expression was analyzed using Western blot. Data are representative of three independent experiments with similar results.

Supplementary Fig. 9

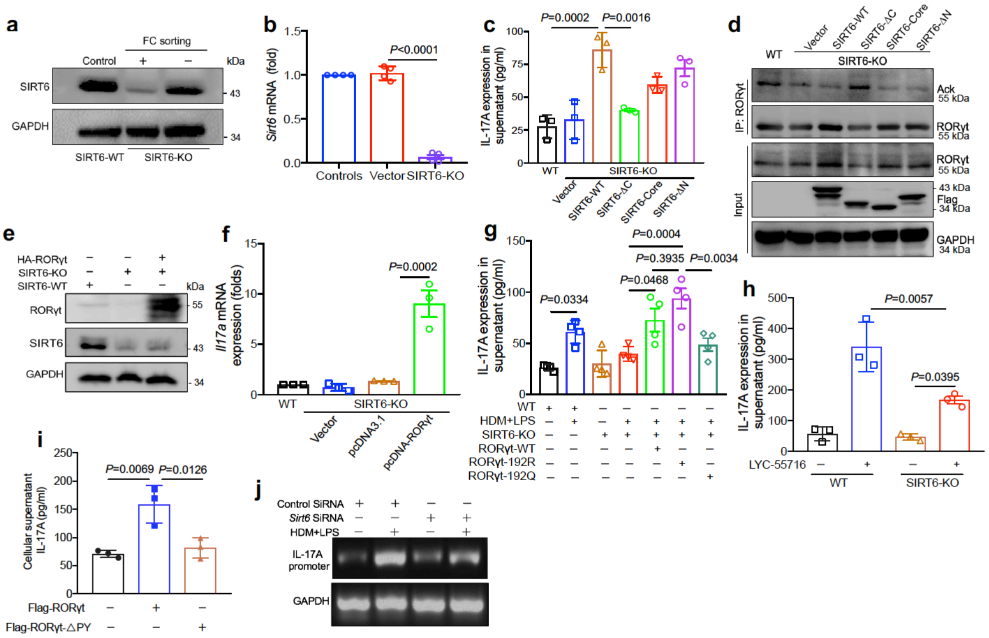

Supplementary Fig. 9 SIRT6-RORγt interaction is essential for IL-17A expression. **a-b** SIRT6-KO HEK293T cells were isolated using Fluorescent cell sorting (FC sorting). Western blot and qRT-PCR analysis of SIRT6 abundance in SIRT6-KO HEK293T cells compared with control. **c** SIRT6-KO HEK293T cells transiently transfected with WT SIRT6 or SIRT6 truncations (SIRT6-Core, SIRT6-ΔN, and SIRT6-ΔC) for 24h and were then treated with HDM/LPS for another 24h. The expression of IL-17A in the supernatant was studied using ELISA analysis. **d** IB analysis of RORγt Ack in SIRT6-KO HEK293T cells transiently transfected with WT SIRT6 or SIRT6 truncations (SIRT6-Core, SIRT6-ΔN, and SIRT6-ΔC), assessed before (input) or after IP with antibody to RORγt and Ack. **e** IB analysis of RORγt and SIRT6 expression in SIRT6-KO cells transfected with RORγt and SIRT6 plasmids. **f** qRT-PCR analysis of *Il17a* expression in SIRT6-KO HEK293T cells transfected with RORγt plasmids. **g** SIRT6-KO HEK293T cells transiently transfected with WT RORγt or RORγt mutant plasmids (K192R and K192Q) for 24h and were then treated with HDM/LPS for another 24h. The expression of IL-17A in the supernatant of above cells was studied by using ELISA analysis. **h** ELISA of IL-17A in supernatant of SIRT6-WT and SIRT6-KO HEK293T

cells incubated with (+) or without (−) RORγt activator LYC-55716 (1 μM) for 72h. **i** ELISA of IL-17A in supernatant of HBE cells transfected with plasmids encoding Flag-RORγt and Flag-RORγt-ΔPY. **j** Effects of *Sirt6* siRNA on HDM/LPS-induced RORγt binding to IL-17A promoter. Data are shown as means ± SEM and three or more independent experiments were performed. Significance was calculated by one-way ANOVA followed by Tukey's post-hoc test for (b, c, f-i).

Supplementary Fig. 10

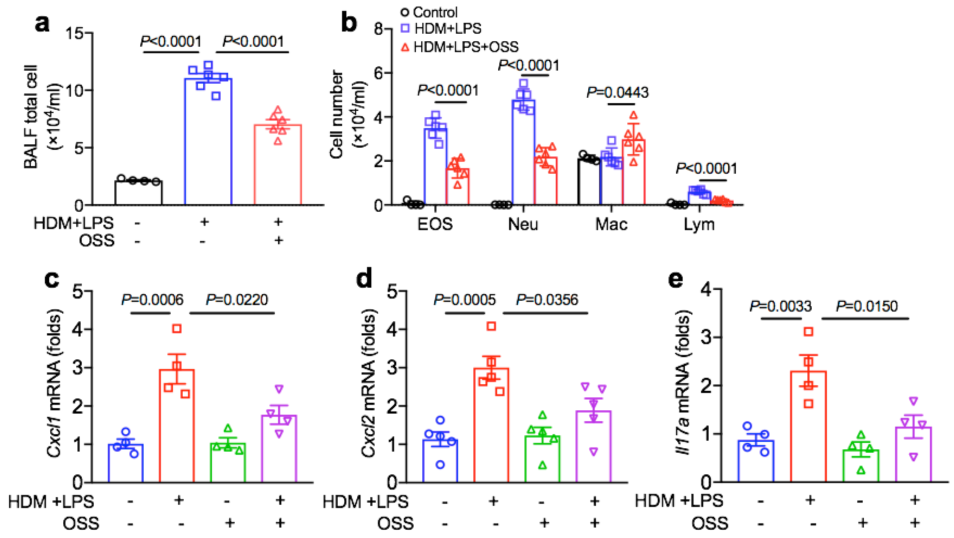

Supplementary Fig. 10 SIRT6 inhibitor OSS reduces airway inflammation in vivo and in vitro. a, b Total BALF cells and differential cell counts from *AE-Sirt6*$^{fl/fl}$ asthmaitc mice treated with or without OSS were assessed (Control n = 4; HDM+LPS n = 6, HDM+LPS+OSS n = 6). **c-e** HBE cells were treated with HDM+LPS and/or OSS for 24h. Inflammatory cytokine expression was determined by RT-PCR. Data are shown as means ± SEM and three or more independent experiments were performed. Significance was calculated by one-way ANOVA followed by Tukey's post-hoc test for (a-e).

Supplementary Fig. 11

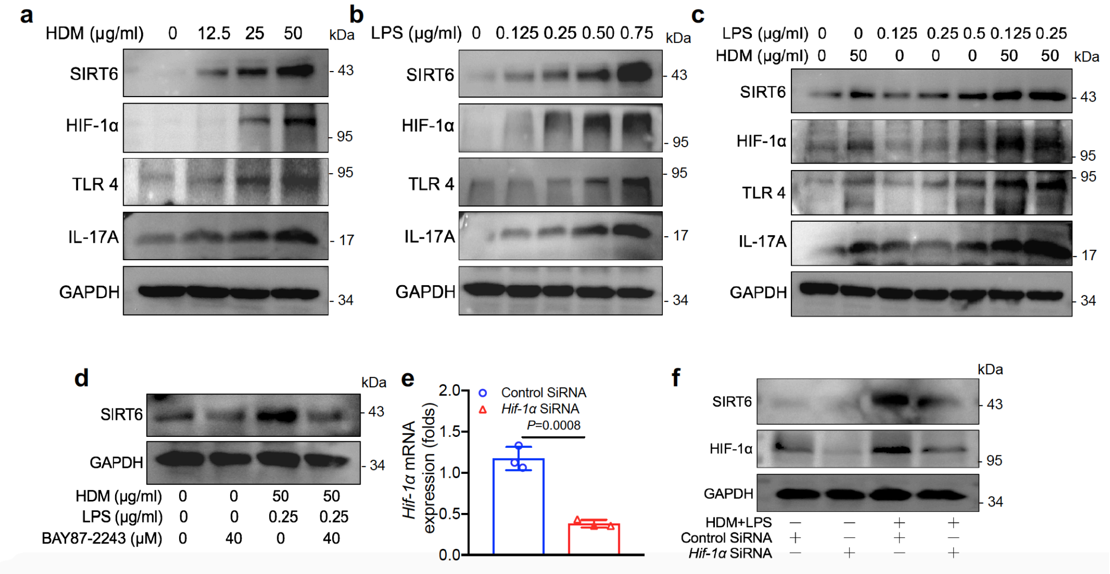

Supplementary Fig. 11 HIF-1α is involved in mediating the HDM/LPS-induced SIRT6 expression. a-c HDM and/or LPS activated SIRT6, IL-17A, HIF-1α, and TLR4 expression in HBE cells via a dose-dependent manner. **d-f** HBE cells were treated with HIF-1α inhibitor BAY87-2243 or transfected with HIF-1α siRNA for 24h and then treated with HDM/LPS for another 24h. SIRT6 expression was determined by Western blot. Data are shown as means ± SEM and three or more independent experiments were performed. Significance was calculated by Two-tailed unpaired Student's *t* test for (e).

Supplementary Fig. 12

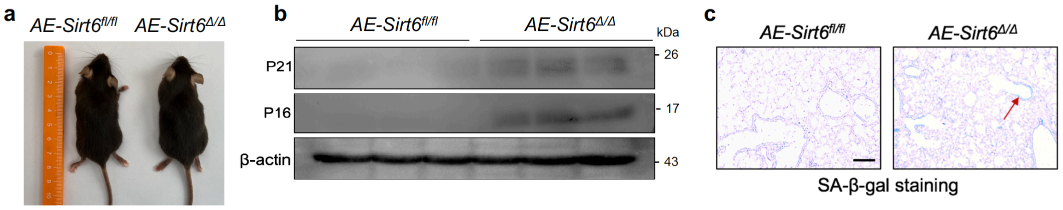

Supplementary Fig. 12 Senescence-related markers in *AE-Sirt6*$^{\Delta/\Delta}$ mice. a Aging-associated gray hair, reduced hair density, and hair loss in *AE-Sirt6*$^{fl/fl}$ and *AE-Sirt6*$^{\Delta/\Delta}$ were assessed. **b-c** Senescence-associated key senescence markers p21, p16, and β-galactosidase (SA-β-Gal) staining in the lung tissues were determined. Scale bars, 100 μm. Three or more independent experiments were performed.

Supplementary Fig. 13

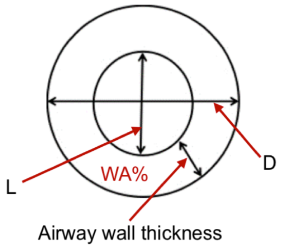

Supplementary Fig. 13 Schematic diagram for measuring the thickness of the airway wall. By measuring the airway diameter (D) and lumen diameter (L), to calculate the percentage of the total cross-sectional area of the airway wall area (WA%) (WA% = $[\pi (D/2)^2 - \pi (L/2)^2]/\pi (D/2)^2 \times 100$).

Table S1. Characteristics of the participants for peripheral blood study

Parameters	Controls (n = 11)	Mild/Moderate asthma (n = 16)	Severe asthma (n = 17)	*P* value
Age, yrs	58.2 ± 1.9	58.7 ± 3.0	58.3 ± 2.9	0.990
Male, n (%)	7 (60)	6 (37.5)	10 (58.8)	0.315
Eos, %	2.34 ± 0.2	5.96 ± 0.3	8.08 ± 0.5	< 0.001
Neu, %	58.18± 1.3	64.13 ± 1.8	72.41 ± 1.60	< 0.001
FEV_1, L	2.61 ± 0.2	2.01 ± 0.1	1.31 ± 0.1	< 0.001
FEV_1/FVC, %	84.68 ± 3.3	86.23 ± 1.7	65.95 ± 2.8	< 0.001
ACT score	N/A	22.6 ± 0.4	17.4 ± 0.3	< 0.001

Data are presented as mean ± SEM, unless otherwise stated.

Significance was calculated by Two-tailed unpaired Student's t test for (ACT score) or one-way ANOVA followed by Tukey's post-hoc test for (Age, Eos, Neu, FEV_1, and FEV_1/FVC) or Chi-square test for (Male). ACT, asthma control test; FVC, forced vital capacity; FEV_1, forced expiratory volume in 1 second; Eos, eosinophils; Neu, neutrophils; N/A, not applicable.

Table S2. Characteristics of the participants for bronchial biopsies study

Groups	Age (ys)	Sex	SIRT6 (%)	IL-17A (pg/ml)	WA (%)	Eos (%)	Clinical diagnosis
Controls	48	M	11	14.63882	0.895	2.11	Lung nodules
	51	M	15	15.04093	0.965	3.27	Lung nodules
	55	M	20	26.83076	0.920	1.78	Lung nodules
	60	M	23	29.6014	1.010	1.86	Lung nodules
	57	F	17	38.42163	1.031	2.29	Lung nodules
Asthmatic patients	51	F	28	39.71741	1.050	5.56	Mild asthma
	58	M	31	44.53372	1.060	6.23	Mild asthma
	50	F	37	66.2088	1.245	5.28	Moderate asthma
	47	F	36	54.84022	1.135	5.27	Moderate asthma
	32	F	30	58.19106	1.335	6.08	Moderate asthma
	65	M	32	43.75776	1.425	10.25	Severe asthma
	48	F	42	84.63459	1.780	8.26	Severe asthma
	66	F	40	57.59177	1.775	7.23	Severe asthma
	48	M	45	84.10876	1.420	11.59	Severe asthma
	32	M	38	70.12499	1.345	6.85	Severe asthma

SIRT6 (%): SIRT6 positive cells in airway epithelium (%); IL-17A (pg/ml): IL-17A expression in bronchoalveolar lavage fluid (BALF); WA(%): Airway wall thickness was expressed as percentage of total airway cross-sectional area; Eos: eosinophils.

Table S3. Primers used for quantitative real time PCR analysis/siRNA sequence

Species/genes	Primer sequence
m-Sirt1 forward	5′-TGATTGGCACCGATCCTCG-3′
m-Sirt1 reverse	5′- CCACAGCGTCATATCATCCAG -3′
m-Sirt2 forward	5′-GCGGGTATCCCTGACTTCC-3′
m-Sirt2 reverse	5′-CGTGTCTATGTTCTGCGTGTAG-3′
m-Sirt3 forward	5′-GAGCGGCCTCTACAGCAAC-3′
m-Sirt3 reverse	5′-GGAAGTAGTGAGTGACATTGGG-3′
m-Sirt4 forward	5′-GATTGACTTTCAGGCCGACAA-3′
m-Sirt4 reverse	5′-GCGGCACAAATAACCCCGA -3′
m-Sirt5 forward	5′-CCAGTTGTGTTGTAGACGAAAGC-3′
m-Sirt5 reverse	5′-TTCCGAAAGTCTGCCATATTTGA-3′
m-Sirt6 forward	5′-AGTGAGGGGCTAATGGGAAC-3′
m-Sirt6 reverse	5′-AACCCACCTCTCTCCCCTAA-3′
m-Sirt7 forward	5′-GCACTTGGTTGTCTACACGG-3′
m-Sirt7 reverse	5′-TGTCCATACTCCATTAGGACCC-3′
m-IL-17A forward	5′-TTTAACTCCCTTGGCGCAAAA-3′
m-IL-17A reverse	5′-CTTTCCCTCCGCATTGACAC-3′
m-IL-17F forward	5′-TGCTACTGTTGATGTTGGGAC-3′
m-IL-17F reverse	5′-AATGCCCTGGTTTTGGTTGAA-3′
m-IL-22 forward	5′-ATGAGTTTTTCCCTTATGGGGAC-3′
m-IL-22 reverse	5′-GCTGGAAGTTGGACACCTCAA-3′
m-CC10 forward	5′-AAA ATC TTG CCAGCT TTC CCC-3′
m-CC10 reverse	5′-ACT GCC CAT TGCCCA AAC AC-3′
m-actin forward	5′-AGTGTGACGTTGACATCCGT-3′
m-actin reverse	5′-GCAGCTCAGTAACAGTCCGC-3′
m-Cxcl-2 forward	5′-TGTCCCTCAACGGAAGAACC-3′
m-Cxcl-2 reverse	5′-CTCAGACAGCGAGGCACATC-3′
m-Cxcl-1forward	5′-CTGGGATTCACCTCAAGAACATC-3′
m-Cxcl-1 reverse	5′-CAGGGTCAAGGCAAGCCTC-3′
m-Teto forward	5′-TGCCACGACCAAGTGACAGCAATG-3′
m-Teto reverse	5′-AGAGACGGAAATCCATCGCTCG-3′
m-MMP3 forward	5′-GGCCTGGAACAGTCTTGGC-3′
m-MMP3 reverse	5′-CTCGCGGCAAGTCTTCAGAG-3′
m-MMP12 forward	5′-GGGCTGCTCCCATGAATGAC-3′
m-MMP12 reverse	5′-CCAGAGTTGAGTTGTCCAGTTG-3′
m-E-cadherin forward	5′-AGCGCAGTCTTACCGAAGG-3′
m-E-cadherin reverse	5′-TCGCTGCTTTCATACTGAACTTT-3′
m-FN1 forward	5′-ATGTGGACCCCTCCTGATAGT-3′
m-FN1 reverse	5′-GCCCAGTGATTTCAGCAAAGG-3′
m-SNAIL forward	5′-CACACGCTGCCTTGTGTCT-3′
m-SNAIL reverse	5′-GGTCAGCAAAAGCACGGTT-3′
m-Muc5acmforward	5′-CTGTGACATTATCCCATAAGCCC-3′
m-Muc5acmreverse	5′-AAGGGGTATAGCTGGCCTGA-3′

m-type I collagen forward	5′-CGGCCCTGCTGGAAACCCTG-3′
m-type I collagen reverse	5′-GGGAGCACCACGTTCACCGG-3′
m-α-SMA forward primer	5′-GCCAGTCGCTGTCAGGAACCCT-3′
m-α-SMA primer	5′-CCAGCCAAGTCCAGACGCAT-3′
m-N-cadherin forward	5′-AGCGCAGTCTTACCGAAGG-3′
m-N-cadherin reverse	5′-TCGCTGCTTTCATACTGAACTTT-3′
h-IL-17A forward	5′-TCCCACGAAATCCAGGATGCC-3′
h-IL-17A reverse	5′-CACAGTGGTCCTTCCAGGTT-3′
h-IL-17F forward	5′-GCTGTCGATATTGGGGCTTG-3′
h-IL-17F reverse	5′-GGAAACGCGCTGGTTTTCAT-3′
h-GAPDH forward	5′-TGTTGCCATCAATGACCCCTT-3′
h-GAPDH reverse	5′-CTCCACGACGTACTCAGCG-3′
h-IL-17A promoter-forward	5′-GCAGCTCTGCTCAGCTTCTA-3′
h-IL-17A promoter-reverse	5′-GGGCTTTTCTCCTTCTGTGG-3′
h-SIRT6 forward	5′-CCCACGGAGTCTGGACCAT-3′
h-SIRT6 reverse	5′-CTCTGCCAGTTTGTCCCTG-3′
h-SNAI1 forward	5′-TCGGAAGCCTAACTACAGCGA-3′
h-SNAI1 reverse	5′-AGATGAGCATTGGCAGCGAG-3′
h-ZEB1 forward	5′-GATGATGAATGCGAGTCAGATGC-3′
h-ZEB1 reverse	5′-ACAGCAGTGTCTTGTTGTTGT-3′
h-FN1 forward	5′-CGGTGGCTGTCAGTCAAAG-3′
h-FN1 reverse	5′-CTCCACGACGTACTCAGCG-3′
h-PDL1 forward	5′- TGGCATTTGCTGAACGCATTT
h-PDL1 reverse	5′-GGGCTTTTCTCCTTCTGTGG-3′
h-Negative control forward	5′-UUCUCCGAACGUGUCACGUTT-3′
h-Negative control reverse	5′-ACGUGACACGUUCGGAGAATT-3′
h-*SIRT6-HOMO-693* forward	5′-UCCAUCACGCUGGGUACAUTT-3′
h-*SIRT6-HOMO-693* reverse	5′-AUGUACCCAGCGUGAUGGATT-3′
h-*SIRT6-HOMO-847* forward	5′-UCAUGACCCGGCUCAUGAATT-3′
h-*SIRT6-HOMO-847* reverse	5′-UUCAUGAGCCGGGUCAUGATT-3′
h-*SIRT6-HOMO-479* forward	5′-GGAAGAAUGUGCCAAGUGUTT-3′
h-*SIRT6-HOMO-479* reverse	5′-ACACUUGGCACAUUCUUCCTT-3′
h-Negative control forward	5′-UUCUCCGAACGUGUCACGUTT-3′
h-Negative control reverse	5′-ACGUGACACGUUCGGAGAATT-3′
h-*HIF1A-HOMO-1614* forward	5′-GCUGGAGACACAAUCAUAUTT-3′
h-*HIF1A-HOMO-1614* reverse	5′-AUAUGAUUGUGUCUCCAGCTT-3′

m: mouse; h: human

Table S4 Key resources table

Reagent	Dilutions	Source	Identifier
Antibodies			
Anti-Collagen Type I	WB (1:800)/IF(1:200)	Proteintech	Cat#67288-1-Ig
Anti-Vimentin	WB (1:5000)	Proteintech	Cat#10366-1-AP
Anti-E-cadherin	WB (1:8000)	Proteintech	Cat#20874-1-AP
Anti-N-cadherin	WB (1:5000)/IF(1:200)	Proteintech	Cat#22018-1-AP
Anti-Smooth muscle actin	WB (1:4000)/IF(1:200)	Proteintech	Cat#14395-1-AP
Anti-IL-17	WB (1:500)/IHC(1:50)	Proteintech	Cat#66148-1-Ig
Anti-Flag	WB (1:1000)	Gen Script	Cat#A00187
Anti-SIRT6	WB (1:200)/IHC, IF(1:50)	Santa Cruz	Cat#sc-517556
Anti-β-actin	WB (1:1000)	Beyotime	Cat#AA128
Anti-Tubulin	WB (1:1000)	Beyotime	Cat#AF0001
Anti-GAPDH	WB (1:10000)	Affinity	Cat#AF7021
Anti-RORγt	WB (1:100)/IF(1:50)	Santa Cruz	Cat#sc-293150
Anti-SIRT5	WB (1:1000)	Abcam	Cat#ab275031
Anti-SIRT1	WB (1:1000)	Abcam	Cat#ab110304
Anti-SIRT3	WB (1:1000)	Abcam	Cat#ab189860
Anti-GST	WB (1:1000)	Santa Cruz	Cat#sc-138
Anti-HA	WB (1:1000)	Abbkine	Cat#ABT2040
Anti-Acetyl Lysine	WB (1:500)	Immunechem	Cat#ICP0380
Anti-Lamin-B1	WB (1:1000)	Abcam	Cat#ab16048
YF-488-Phalloidin	IF (1:200)	UElandy	Cat#YP0059S
Alexa Fluor 488	IF (1:1000)	Beyotime	Cat#A0423
Alexa Flour 555	IF (1:1000)	Beyotime	Cat#A0460
Anti-SCGB1A1/CC10	WB (1:500)/IF(1:50)	Proteintech	Cat#26909-1-AP
Anti-F4/80	IF (1:50)	Proteintech	Cat#29414-1-AP
Anti-CD31	IF (1:50)	Proteintech	Cat#11265-1-AP
Duolink® PLA reagent		MERCK	Cat#DUO92102
Mouse IL-17/IL-17A Antibody	WB (1:1000)	R&D	Cat#MAB421-SP
Anti-CD3 (APC/Cyamine7)	0.25 µg per million cells	Biolegend	Cat#100222
Anti-CD4 (FITC)	0.25 µg per million cells	Biolegend	Cat#100405
Anti-F4/80 (APC)	0.25 µg per million cells	Biolegend	Cat#100405
Anti-CD49b (PE)	0.25 µg per million cells	Biolegend	Cat#108907
Anti-CD11c (PE)	0.25 µg per million cells	Biolegend	Cat#117308

Anti-CD19 (Percp/Cyamine7)	0.25 µg per million cells	Biolegend	Cat#115533
Anti-HIF-1α	WB (1 :500)	Proteintech	Cat#20960-1-AP
Anti-Prosurfactant Protein C	WB (1:1000)/IF (1:500)	Abcam	Cat#ab90716
Anti-HIF-1α	WB (1 :1000)	Abcam	Cat#ab51608
Anti-TLR4	WB (1:200)	Santa Cruz	Cat# sc52962
Anti-P21 Waf1/Cip1	WB (1:1000)	CST	Cat#64016
Anti-P16	WB (1:500)	Santa Cruz	Cat#sc1661
Anti-IL17	WB (1:50)/IF(1:50)	SantaCruz	Cat#sc374218
Anti-F4/80	IF (1:50)	Proteintech	Cat#28463-1-AP
**Chemicals, recombinant proteins**			
TMP920		MCE	Cat#1421837-45-7
Recombinant Murine IL-17A		Peprotech	Cat#210-17
Recombinant Human IL-17A		Peprotech	Cat#210-17
OSS_128167		MCE	Cat#HY-10745
LYC-55716		Selleck	Cat#S8860
HDM		GREER	Cat#XPB82D3A25
LPS		SIGMA	Cat#L2880-25MG
MDL-800		APEXBIO	Cat#B8384
NAM		Beyotime	Cat#P1112
DAPI		Beyotime	Cat#C1005
BAY87-2243		Beyotime	Cat#SC1193
Protein G Beads		Beyotime	Cat#P2055
**Critical commercial assays**			
ChIP Assay Kit		Beyotime	Cat#P2078
Dual Luciferase Reporter Assay System Kit		Promega	Cat#E1910
GST-tag Protein Purification Kit		Beyotime	Cat#P2262
Nuclear and Cytoplasmic Protein Extraction Kit		Beyotime	Cat#P0027
Human IL-17A ELISA Kit		Elabscience	Cat#E-EL-H5812c
Mouse IL-17A ELISA Kit		Elabscience	Cat#E-EL-M0047c
Mouse GROα/CXCL1 ELISA Kit		Elabscience	Cat#E-EL-M0018c
Human GROα/CXCL1 ELISA Kit		Elabscience	Cat#E-EL-H0045c
Mouse GROβ/CXCL2 ELISA Kit		Elabscience	Cat#E-EL-M0019c
Critical commercial assays			
Human GROβ/CXCL2 ELISA Kit		Elabscience	Cat#E-EL-H1904c
TB Green® Premix Ex Taq™		Takara	Cat#RR420A
Prime Script™ RT reagent Kit		Takara	Cat#RR047A
Express Cast PAGE Gel Preparation kit		NCM Biotech	Cat#P2012

Senescence β-Galactosidase Staining Kit	Beyotime	Cat#C0602
Mouse IL-13 ELISA Kit	Elabscience	Cat#E-EL-M0727c
Mouse IL-5 ELISA Kit	Elabscience	Cat#E-EL-M0722c
Deposited Data		
Raw and analyzed RNA sequencing data	This manuscript	N/A
Raw data	This manuscript	N/A
Mass spectrometry (MS)	This manuscript	N/A
Experimental Models: Cell Lines		
HEK293T	ATCC	CRL-11268
Human bronchial epithelium cells	ATCC	CRL-2741
Human vein endothelial cells	ATCC	CRL-2480
Human smooth muscle cells	Procell	CP-HPP3
Human SIRT6 KO cells	This manuscript	N/A
Primary mouse lung fibroblast cells	This manuscript	N/A
Recombinant DNA		
pcDNA3.1-HA	Yubo Biotechnology, China	N/A
pGEX-4T-2-HA-RORγt,	Yubo Biotechnology, China	GEX-HA-6097
pCAGPuro-RORγt-HA	Yubo Biotechnology, China	6097-HA
RORγt-D-PPLY	Yubo Biotechnology, China	6097-D-PPLY
RORγt-K192Q	Yubo Biotechnology, China	6097-K192Q
RORγt-K192R	Yubo Biotechnology, China	6097-K192R
GST-SIRT6	Yubo Biotechnology, China	GEX-23408
Software and Algorithms		
GraphPad Prism 8.0	https://www.graphpad.com	N/A
Image J	https://imagej.net/Welcome	N/A
Real-Time PCR Systems	Applied Biosystem	N/A
Cytoscape (v.3.5.1)	https://www.cytoscape.org/	N/A
R package clusterProfiler	Yu et al., 2012	N/A
R package GEOquery	Davis and Meltzer, 2007	N/A
**Mice**		
C57BL/6 wild-type (WT) mice	GemPharmatech Co., Ltd., China	N/A
Scgb1a1-rtTA/(tetO)7-Cre transgenic mice	GemPharmatech Co., Ltd., China	N/A
Sirt6$^{fl/fl}$ mice	The Jackson Laboratory	N/A
