# Supplementary material for: Epithelial SIRT6 governs IL-17A pathogenicity and drives allergic airway inflammation and remodeling
Source: Nat Commun. 2023 Dec 22;14:8525. doi: 10.1038/s41467-023-44179-x (PMC10746710; doi:10.1038/s41467-023-44179-x)
Supplement: Supplementary file 3 — Description of Additional Supplementary Files [file 41467_2023_44179_MOESM3_ESM.pdf]

Description of Additional Supplementary Files

Supplementary Data 1: RNA-Seq data.

Supplementary Data 2: Immunoprecipitation in combination with mass spectrometry (IP-MS) data.

Supplementary Data 3: Identification of acetylation site by LC-MS/MS analysis.
