# Supplementary material for: Epithelial SIRT6 governs IL-17A pathogenicity and drives allergic airway inflammation and remodeling
Source: Nat Commun. 2023 Dec 22;14:8525. doi: 10.1038/s41467-023-44179-x (PMC10746710; doi:10.1038/s41467-023-44179-x)
Supplement: Supplementary file 7 — Reporting Summary [file 41467_2023_44179_MOESM7_ESM.pdf]

nature portfolio

Corresponding author(s): Tianwen Lai, Jing Tang, Xiao Gao

Last updated by author(s): Nov 6, 2023

Reporting Summary

Nature Portfolio wishes to improve the reproducibility of the work that we publish. This form provides structure for consistency and transparency in reporting. For further information on Nature Portfolio policies, see our Editorial Policies and the Editorial Policy Checklist.

Statistics

For all statistical analyses, confirm that the following items are present in the figure legend, table legend, main text, or Methods section.

n/a	Confirmed	
☐	☒	The exact sample size (*n*) for each experimental group/condition, given as a discrete number and unit of measurement
☐	☒	A statement on whether measurements were taken from distinct samples or whether the same sample was measured repeatedly
☐	☒	The statistical test(s) used AND whether they are one- or two-sided<br>*Only common tests should be described solely by name; describe more complex techniques in the Methods section.*
☐	☒	A description of all covariates tested
☒	☐	A description of any assumptions or corrections, such as tests of normality and adjustment for multiple comparisons
☐	☒	A full description of the statistical parameters including central tendency (e.g. means) or other basic estimates (e.g. regression coefficient) AND variation (e.g. standard deviation) or associated estimates of uncertainty (e.g. confidence intervals)
☐	☒	For null hypothesis testing, the test statistic (e.g. *F*, *t*, *r*) with confidence intervals, effect sizes, degrees of freedom and *P* value noted<br>*Give P values as exact values whenever suitable.*
☒	☐	For Bayesian analysis, information on the choice of priors and Markov chain Monte Carlo settings
☒	☐	For hierarchical and complex designs, identification of the appropriate level for tests and full reporting of outcomes
☐	☒	Estimates of effect sizes (e.g. Cohen's *d*, Pearson's *r*), indicating how they were calculated

Our web collection on statistics for biologists contains articles on many of the points above.

Software and code

Policy information about availability of computer code

Data collection	No software was used
Data analysis	GraphPad Prism 8.0, Image J, ELISA Calc-v0.1, Flow Jo, Cytoscape (v.3.5.1).

For manuscripts utilizing custom algorithms or software that are central to the research but not yet described in published literature, software must be made available to editors and reviewers. We strongly encourage code deposition in a community repository (e.g. GitHub). See the Nature Portfolio guidelines for submitting code & software for further information.

Data

Policy information about availability of data

All manuscripts must include a data availability statement. This statement should provide the following information, where applicable:

- Accession codes, unique identifiers, or web links for publicly available datasets
- A description of any restrictions on data availability
- For clinical datasets or third party data, please ensure that the statement adheres to our policy

The data that support the findings of this study are available within the article and its Supplementary Information or from the corresponding authors on reasonable request.Raw data are shown in data file S1 and file S2. RNA-seq data that support the findings of this study have been deposited in Genome Sequence Archive with the accession codes PRJNA1003640 (https://submit.ncbi.nlm.nih.gov/).

Research involving human participants, their data, or biological material

Policy information about studies with human participants or human data. See also policy information about sex, gender (identity/presentation), and sexual orientation and race, ethnicity and racism.

Reporting on sex and gender	In our study, asthmatic patients between 18 and 65 years old were included. Exclusion criteria were combined respiratory diseases other than asthma such as chronic obstructive pulmonary disease (COPD), and lung cancer. Patients who had lung nodules and underwent surgery were used as normal control. Spirometry was done by a turbine spirometry device (Jaeger, Germany) according to the GINA guideline. The sex/gender was considered in our study design.
Reporting on race, ethnicity, or other socially relevant groupings	None
Population characteristics	The mean age of mild-moderate asthma and severe group was 58.7 ± 3.0y and 58.3 ± 2.9y, respectively. The mean age of control group was 58.2±1.9y. The information for Age and Gender was not statistically significant between disease and control groups. Detailed clinical characteristics are summarized in Supplementary Table 1 and Table 2.
Recruitment	The diagnosis of asthma and disease severity was based on the Global Initiative on Asthma (GINA) guidelinesl. Asthmatic patients between 18 and 65 years old were included. Exclusion criteria were combined respiratory diseases other than asthma such as chronic obstructive pulmonary disease (COPD), and lung cancer. Patients who had lung nodules and underwent surgery were used as normal control. Peripheral blood mononuclear cells (PBMC) were isolated from peripheral blood samples of study subjects using density gradient centrifugation according to manufacturer instructions. Bronchial biopsies and BALF were obtained from patients undergoing bronchoscopy for diagnostic purposes. Clinical information is summarized in Table 51 and S2.
Ethics oversight	The study subjects were recruited from two medical centers and the study protocols were approved by the Medical Ethics Committee of Affiliated Hospital of Guangdong Medical University and the Second Hospital Zhejiang University (PJKT2022-079, ChiCTR-OOC-15006345). Written informed consent was obtained from all patients included in the study.

Note that full information on the approval of the study protocol must also be provided in the manuscript.

Field-specific reporting

Please select the one below that is the best fit for your research. If you are not sure, read the appropriate sections before making your selection.

- [x] Life sciences
- [ ] Behavioural & social sciences
- [ ] Ecological, evolutionary & environmental sciences

For a reference copy of the document with all sections, see nature.com/documents/nr-reporting-summary-flat.pdf

Life sciences study design

All studies must disclose on these points even when the disclosure is negative.

Sample size	In general, no calculations were done to determine sample size. Sample size was chosen based on literature and variability observed in previous experience in the laboratory. In this study, sample size was determined based on the standards for cell experiments attempting to have a minimum of N = 3 biological independent samples and animal experiments attempting to have a minimum of N = 5 biological independent samples with sufficient reproducibility. Quantitative analysis of confocal images included replicates of different field scans per independent sample. The determination of sample size chosen in our study did not include the high-throughput experiments with big dataset from public database. The details on the sample size were included in each figure legend.
Data exclusions	No data were excluded from the analyses.
Replication	In this study, each result described in the paper is based on at least three independent biological replicates but very often an experiment is based on more than three experiments. Figure legends indicate the number of independent experiments performed in each analysis.
Randomization	For all the experiments, samples were randomly allocated to experimental and control groups.
Blinding	For all the animal and cell experiments, the investigators were blinded to the group allocations during the measurements and data analysis, and the samples were tested in a randomized order. The blinding was not relevant in obtaining the PBMC, biopsy tissues, and BALF from control and asthmatic patients.

Reporting for specific materials, systems and methods

We require information from authors about some types of materials, experimental systems and methods used in many studies. Here, indicate whether each material, system or method listed is relevant to your study. If you are not sure if a list item applies to your research, read the appropriate section before selecting a response.

Materials & experimental systems

n/a	Involved in the study
☐	☒ Antibodies
☐	☒ Eukaryotic cell lines
☒	☐ Palaeontology and archaeology
☐	☒ Animals and other organisms
☐	☒ Clinical data
☒	☐ Dual use research of concern
☒	☐ Plants

Methods

n/a	Involved in the study
☒	☐ ChIP-seq
☐	☒ Flow cytometry
☒	☐ MRI-based neuroimaging

Antibodies

Antibodies used

1. Anti-Collagen Type I Proteintech Cat#67288-1-Ig
2. Anti-Vimentin Proteintech Cat#10366-1-AP
3. Anti-E-cadherin Proteintech Cat#20874-1-AP
4. Anti-N-cadherin Proteintech Cat#22018-1-AP
5. Anti-Smooth muscle actin Proteintech Cat#14395-1-AP
6. Anti-IL-17 Proteintech Cat#66148-1-Ig
7. Anti-Flag Gen Script Cat#A00187
8. Anti- -actin Beyotime Cat#AA128
9. Anti-Tubulin Beyotime Cat#AF0001
10. Anti-GAPDH Affinity Cat#AF7021
11. Anti-ROR t Santa Cruz Cat#sc-293150
12. Anti-SIRT5 Abcam Cat#ab275031
13. Anti-SIRT1 Abcam Cat#ab 110304
14. Anti-SIRT3 Abcam Cat#ab189860
15. Anti-GST Santa Cruz Cat#sc-138
16. Anti-HA Abbkine Cat#ABT2040
17. Anti-Acetyl Lysine Immunechem Cat#ICP0380
18. Anti-Lamin-B1 Abcam Cat#ab16048
19. YF-488-Phalloidin Bioscience CatftYP0059S
20. Alexa Fluor 488 Beyotime Cat#A0423
21. Alexa Flour 555 Beyotime Cat#A0460
22. Anti-SCGB1A1 Proteintech Cat#26909-1-AP
23. Anti-F4/80 Proteintech Cat#29414-1-AP
24. Anti-CD31 Proteintech Cat#11265-1-AP
25. Duolink® PLA reagent MERCK Cat#DUO92102
26. Mouse IL-17/IL-17A Antibody R D Cat#MAB421-SP
27. Anti-CD3 (APC/Cyamine7) Biolegend Cat#100222
28. Anti-CD4 (FITC) Biolegend Cat#100405
29. Anti-F4/80 (APC) Biolegend Cat#123116
30. Anti-CD49b (PE) Biolegend Cat#108907
31. Anti-CD11c (PE) Biolegend Cat#117308
32. Anti-CD19 (Percp/Cyamine7) Biolegend Cat#115533
33. Anti-HIF-1 Proteintech Cat#20960-1-AP
34. Anti-Prosurfactant Protein C Abcam Cat#ab90716
35. Anti-HIF-1 Abcam Cat#ab51608
36. Anti-TLR4 Santa Cruz Cat# sc52962
37. Anti-P21 Waf1/Cip1 CST Cat#64016
38. Anti-P16 Santa Cruz Cat#sc1661
39. Anti-F4/80 Proteintech Cat#28463-1-AP
40. Anti-IL17 SantaCruz Cat#sc374218
41. Anti-SIRT6 Santa Cruz Cat#sc-517556

Validation

1. https://www.ptgcn.com/products/Collagen-I-Antibody-67288-1-Ig.htm
2. https://www.ptgcn.com/products/VIM-Antibody-10366-1-AP.htm
3. https://www.ptgcn.com/products/E-cadherin-Antibody-20874-1-AP.htm
4. https://www.ptgcn.com/products/N-cadherin-Antibody-22018-1-AP.htm
5. https://www.ptgcn.com/products/ACTA2-Antibody-14395-1-AP.htm
6. https://www.ptgcn.com/products/IL17A-Antibody-66148-1-Ig.htm
7. https://www.genscript.com/search?q=A00187&search=Search

8.https://www.beyotime.com/product/AA128.htm

9.https://www.beyotime.com/product/AF0001.htm

10.https://www.affbiotech.cn/goods-6289-AF7021-GAPDH_Antibody.html

11. https://www.scbt.com/p/rorgamma-antibody-27-92?requestFrom=search

12.https://www.abcam.cn/products/primary-antibodies/sirt5-antibody-epr23787-116-bsa-and-azide-free-ab275031.html
13. https://www.abcam.cn/products/primary-antibodies/sirt1-antibody-19a7ab4-ab110304.html
14.https://www.abcam.cn/products/primary-antibodies/sirt3-antibody-ab189860.html
15.https://www.scbt.com/p/gst-antibody-b-14?requestFrom=search
16.https://www.abbkine.com/?s_type=productsearch&s=ABT2040
17 .https ://i mmu nechem .com/product/acetyl-lysine-a ntibody /
18.https://www.abcam.cn/products/primary-antibodies/lamin-bl-antibody-nuclear-envelope-marker-abl6048.html
19.http://www.uelandy.com/productDe_47.html
20.https ://www.beyotime.com/product/ A0423.htm
21.https ://www.beyotime.com/product/ A0460.htm
22 .https://www.ptgcn.com/products/Uteroglobin-Antibody-26909-1-AP .htm
23.https://www.ptgcn.com/products/F4-80-Anti body-29414-1-AP .htm
24.https://www.ptgcn.com/products/PECAM 1-Antibody-11265-1-AP .htm
25.https ://www.sigmaaldrich.cn/CN/zh/search/duo92102?
focus=products&page=l&perpage=30&sort=relevance&term=duo92102&type=product
26.https ://www.rndsystems.com/cn/products/mouse-il-17-il-17a-anti body-50104_mab421
27.https://www.biolegend.com/en-us/products/apc-cyanine7-anti-mouse-cd3-antibody-6068
28. https ://www. biol egen d. com/ e n-u s/p rod u cts/fitc-a nti-m ou se-cd4-a nti body-2 48
29. https ://www. biol egen d. com/ e n-u s/p rod ucts/a pc-a nti-m ou se-f 4-80-a nti body-4071
30. https ://www. biol egen d. com/ e n-u s/p rod u cts/pe-a nti-m o use-cd49 b-pa n-n k-ce 11 s-a nti body-2 34
31.https://www.biolegend.com/en-us/products/pe-anti-mouse-cdllc-antibody-1816
32. https ://www. biol egen d. com/ e n-u s/p rod u cts/percp-cya n i neS-5-a nti-m o use-cd 19-a nti body-4 2 61
33.https://www.ptgcn.com/products/H I FIA-Antibody-20960-1-AP .htm
34. https ://www .a bca m .cn/p rod ucts/p rim a ry-a nti bodies/ prosu rfa cta nt -protei n-c-a nti body-a b90716. htm I
35.https://www.abcam.cn/p 「oducts/primary-antibodies/hif-1-a lpha-anti body-ep 12 lSy-a bS 1608. html
36.https://www.scbt.com/p/tl r4-antibody-76b35 7-1 ?requestFrom=search
37 .https://www.cellsignal.cn/products/pri mary-anti bodies/p21-wafl-cip 1-antibody /64016?site-sea rchtype=
Products&N=4294956287&Ntt=64016&fromPage=plp&_requestid=1808893
38.https://www .scbt.com/p/p 16-anti body-f-12 ?requestFrom=search
39.https://www.ptgcn.com/products/F4-80-Anti body-28463-1-AP .htm
40.https://www.scbt.com/p/i 1-17-antibody-g-4 ?requestFrom=sea rch
41. https ://www.scbt.com/ p/ si rt6-a nti body-6c9-d 10-d 3 ?request F 「om=search

Eukaryotic cell lines

Policy information about cell lines and Sex and Gender in Research

Cell line source(s)	Antibody informations are summarized in Table S7.<br>HEK293T ATCC CRL-11268<br>Human bronchial epithelium cells ATCC CRL-2741<br>Human vein endothelial cells ATCC CRL-2480<br>Human smooth muscle cells Procell CP-HPP3<br>Human SIRT6 KO cells This manuscript N/A<br>Primary mouse lung fibroblast cells This manuscript N/A
Authentication	Cell identities were confirmed routinely by western blot and immunoflourescence marker expressions.
Mycoplasma contamination	Cell lines were routinely tested for mycoplasma contamination by mycoplasma detection kit and confirmed that they were negative fo 「mycoplasma contamination.
Commonly misidentified lines (See ICLAC register)	No commonly misidentified cell lines were used.

Animals and other research organisms

Policy information about studies involving animals; ARRIVE guidelines recommended for reporting animal research, and Sex and Gender in Research

Laboratory animals	C57BL/6 wild-type (WT) mice were purchased from the GemPharmatech Co., Ltd. (Nan ﾌ ing, China). Sirt6flox/flox (Sirt6fl/fl) on the C57BL/6 background were purchased from The Jackson Laboratory. We generated Scgblal-rtTA/(tet0)7-Cre/Sirt6fl/fl (designated as AE-Sirt66/ 6) mice, in which SIRT6 was specifically depleted f 「om airway epithelium cells, by mating Sirt6fl/fl mice with ScgblalrtTA/(tet0)7-Cre transgenic mice (C57BL/6 background). Age- and sex-matched Sirt6fl/fl mice were used as controls in the experiments. To induce the expression of Cre, 6-week-old mice were fed with doxycycline (DOX) in drinking water (2 mg/ml) for 20 days before establishing the model of allergen-induced airway inflammation, and the mice were kept with DOX at all the time until they were sacrificed. All animals were age-matched and sex-matched, and then randomized into different groups
Wild animals	No wild animals were used in this study.

Reporting on sex	We included both male and female mice in our study and reported them differently in the figures. We did not find any sex
Field-collected samples	No sample was collected from the field.
Ethics oversight	All mice were maintained in specific pathogen-free animal facilities at the Animal Care Facility of Guangdong Medical University. Experiments were conducted according to the Declaration of Helsinki conventions for the use and care of animals and were approved by the Animal Care and Use Committee at Guangdong Medical University.

Note that full information on the approval of the study protocol must also be provided in the manuscript.

Clinical data

Policy information about clinical studies

All manuscripts should comply with the ICMJEguidelines for publication of clinical research and a completedCONSORT checklist must be included with all submissions.

Clinical trial registration	N/A
Study protocol	N/A
Data collection	N/A
Outcomes	N/A

Plants

Seed stocks	N/A
Novel plant genotypes	N/A
Authentication	N/A

Flow Cytometry

Plots

Confirm that:

- [x] The axis labels state the marker and fluorochrome used (e.g. CD4-FITC).
- [x] The axis scales are clearly visible. Include numbers along axes only for bottom left plot of group (a 'group' is an analysis of identical markers).
- [x] All plots are contour plots with outliers or pseudocolor plots.
- [x] A numerical value for number of cells or percentage (with statistics) is provided.

Methodology

Sample preparation	Lung tissues of mice were prepared into cell suspension, and appropriate amount of surface marker antibodies were added according to the instructions, and incubated for 30 min on ice, protected from light. Flow cytometry data were obtained using Fortessa flow cytometry (BD FACSCanto Ⅱ) and analyzed using FlowJo software (Becton Dickinson). Single-cell suspensions were stained with combinations of the following antibodies (Biolegend) : anti-CD3 (APC/Cyamine7), anti-CD4 (FITC), anti-F4/80 (APC), anti-CD49b (PE), anti-CD11c (PE), and anti-CD19 (Percp/Cyamine7).
Instrument	Olympus FV3000 (Japan) scanning system was used to acquire immunofluorescence image.<br>ChemiScope S6 (Clinx Science Instruments Co., Ltd,Shanghai) was used to acquire western blot images.<br>Light Cycler R 480 II (Roche, Switzerland) were used to acquire qRT-PCR results.<br>Optical absorption full wavelength microplate reader (ReadMax 1200,Flash,Shanghai)was used to acquire ELISA assay<br>Fortessa flow cytometry (BD FACSCanto Ⅱ , Becton Dickinson,USA) was used to acquire Flow cytometry results .
Software	Immunofluorescence images were contrasted, overlaid, and analyzed with FV31S-SW software.<br>Western blot images were contrasted and analyzed with CS mini chemiluminescence imaging acquisition and processing software and Image J software.<br>Real-Time PCR results were analyzed with Graphpad Prism 8.0.<br>ELISA results were analyzed with ELISA Calc- v 0.1.

	Flow cytometry results were analyzed with FlowJo software (Becton Dickinson,USA).
Cell population abundance	Cell purities were validated using flowcytometry measurements and were >99%.
Gating strategy	Please Supplementary Figure 4S for representative gating strategies. After cells clustered or with severely morphological changes were removed by grouping through forward scatter (FSC) and side scatter (SSC), the remaining cells were first gated by CD45+. The cells selected as CD45+ were then gated by the transdifferentiation marker (CD3+ for example) to screen out the CD8+, CD4+, CD49b, CD19b or CD11c.

☒ Tick this box to confirm that a figure exemplifying the gating strategy is provided in the Supplementary Information.
