# Supplementary figures and images for: Epithelial SIRT6 governs IL-17A pathogenicity and drives allergic airway inflammation and remodeling
Source: Nat Commun. 2023 Dec 22;14:8525. doi: 10.1038/s41467-023-44179-x (PMC10746710; doi:10.1038/s41467-023-44179-x)

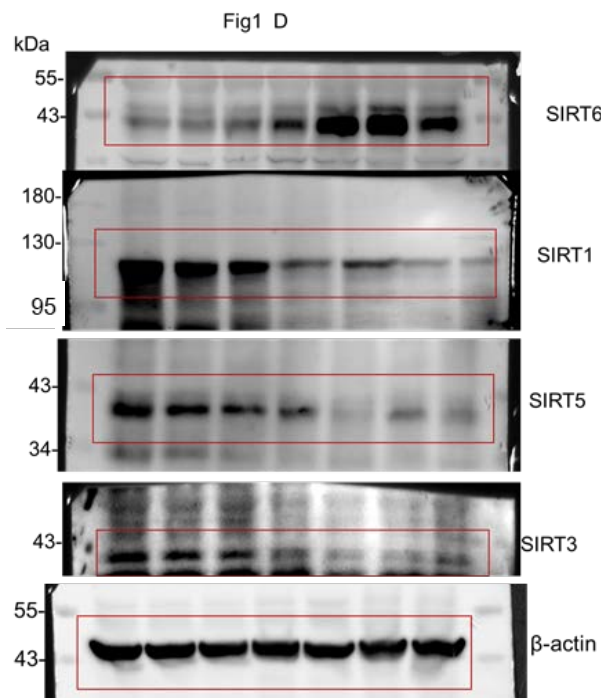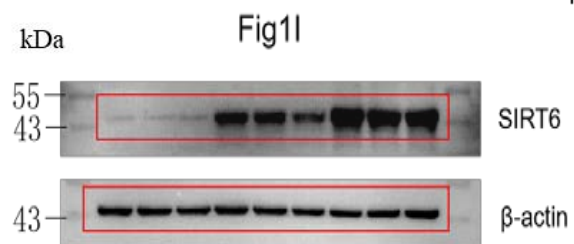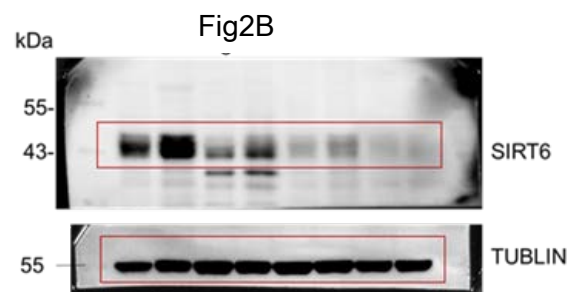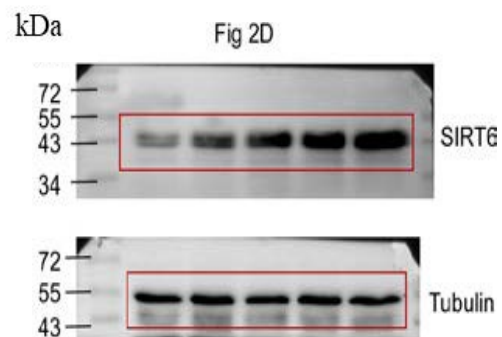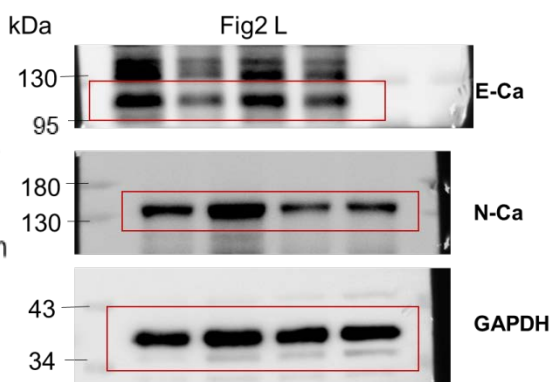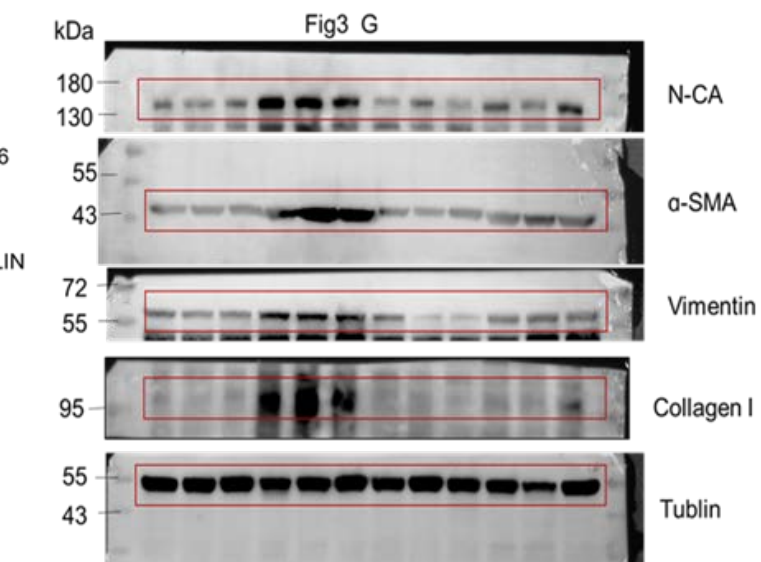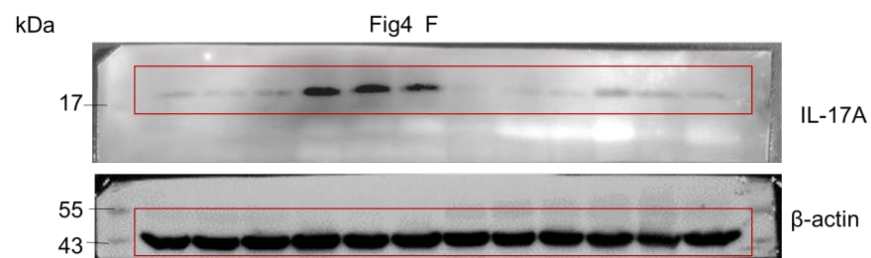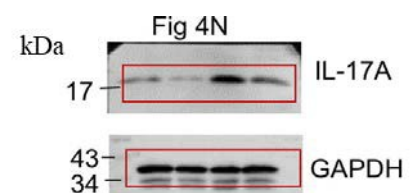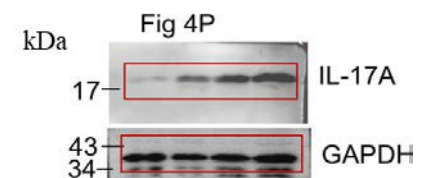

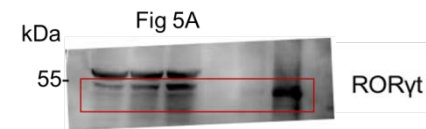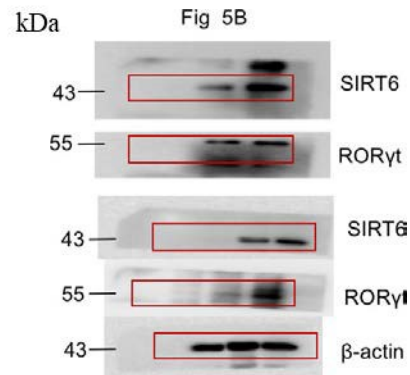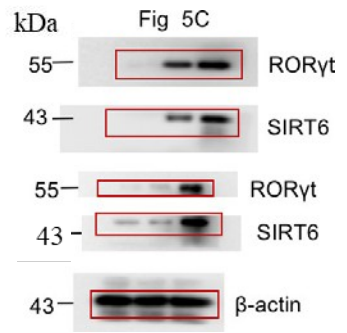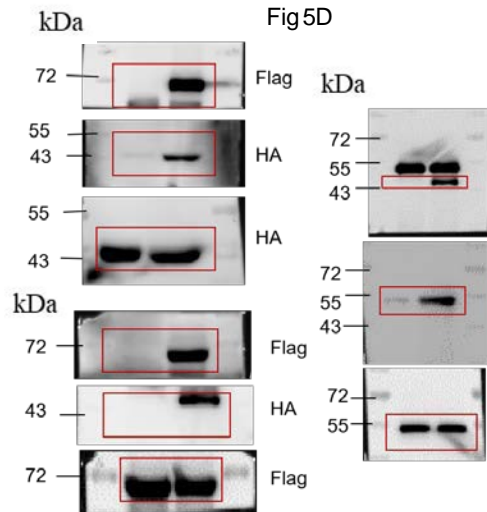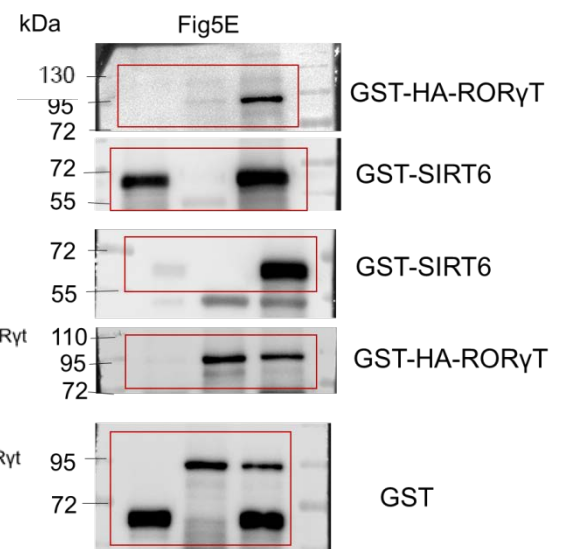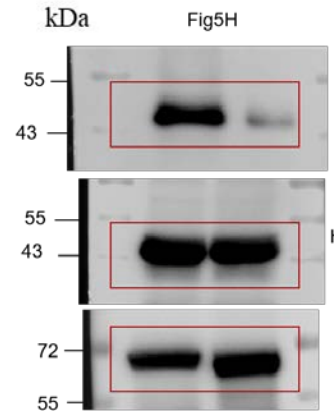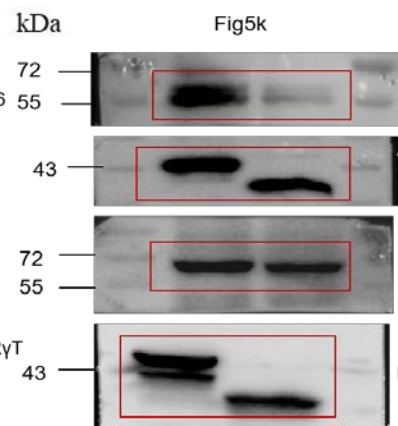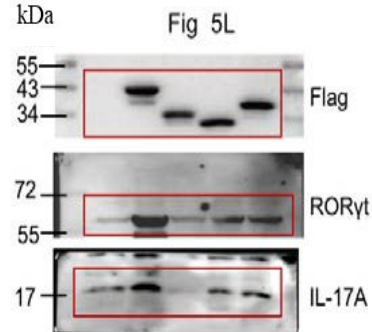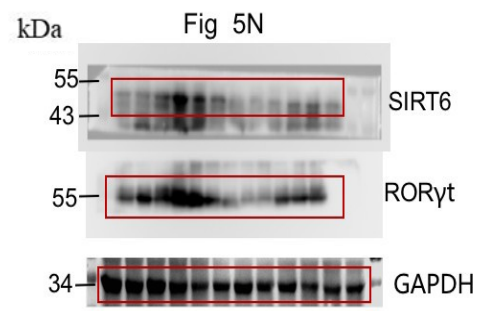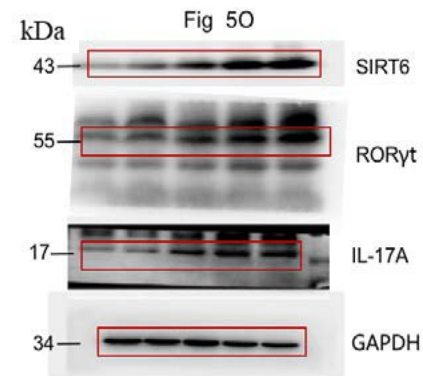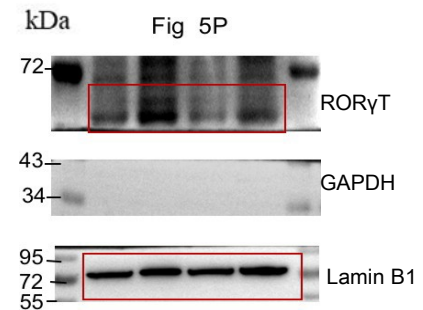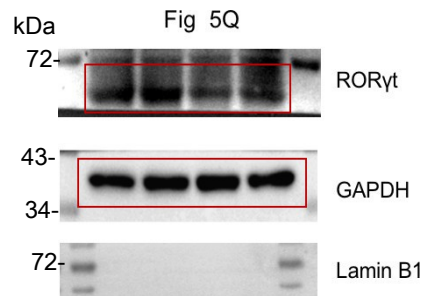

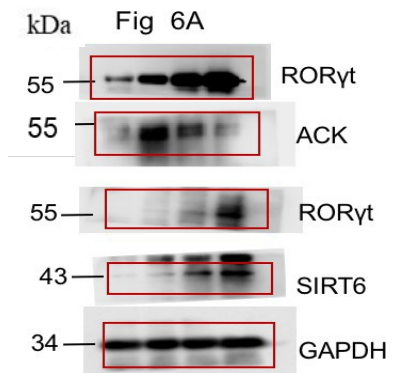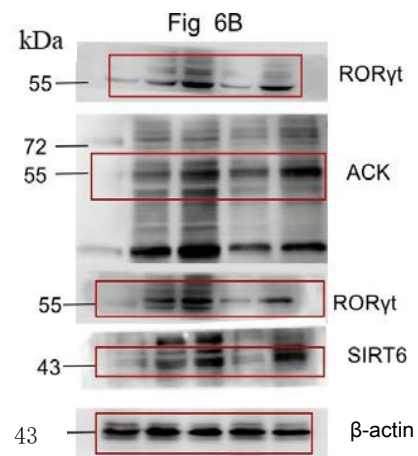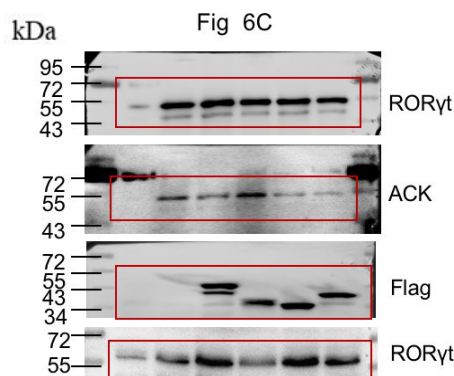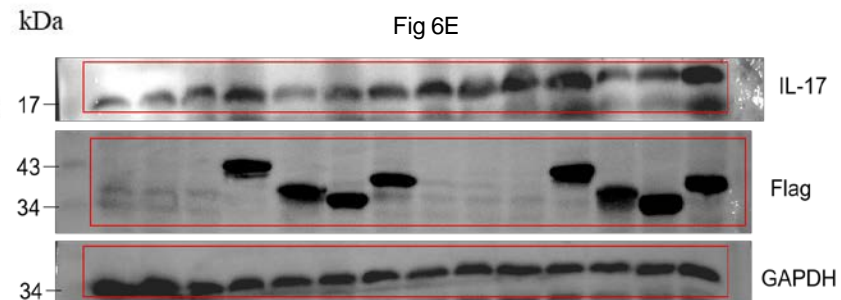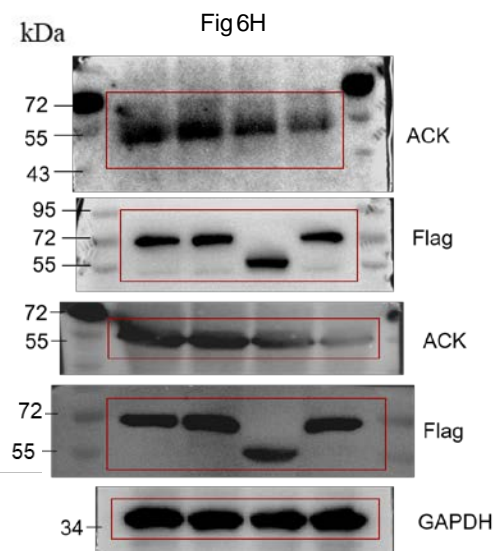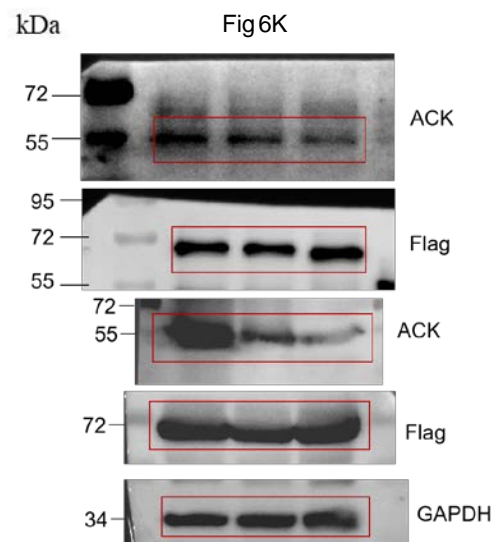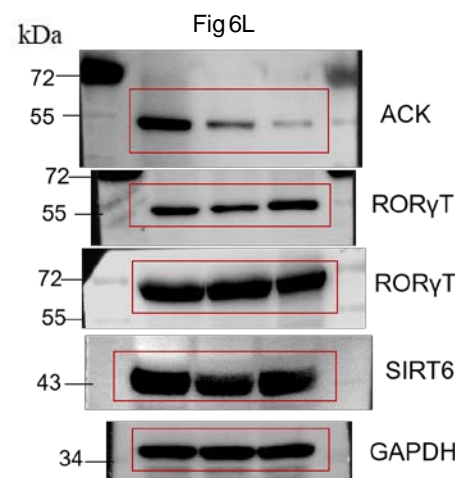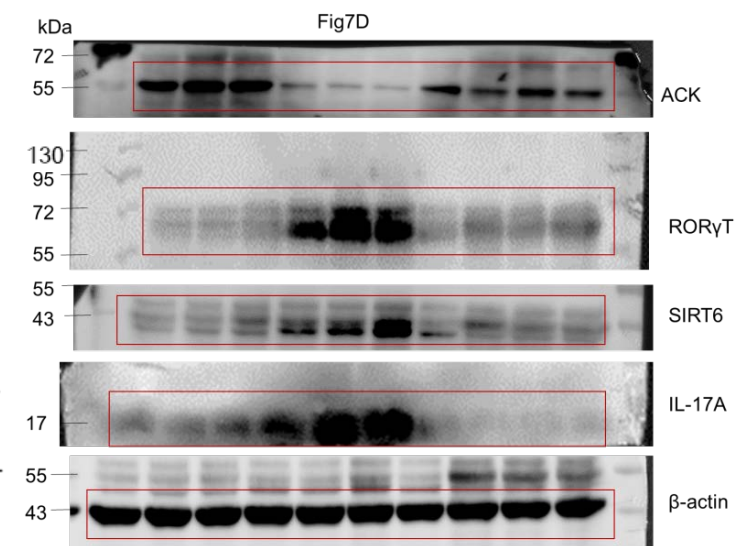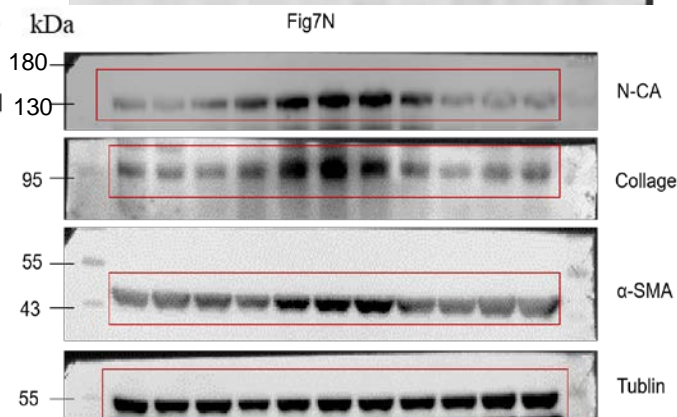

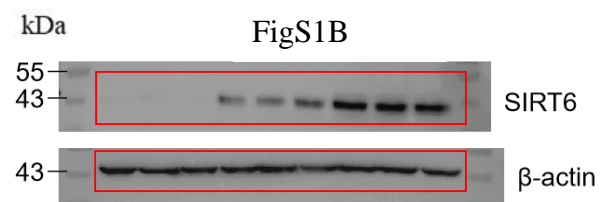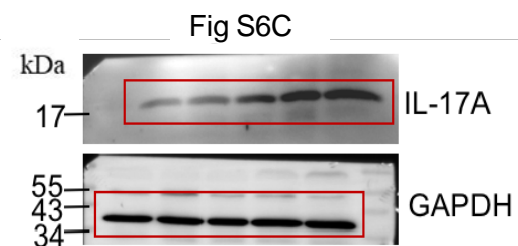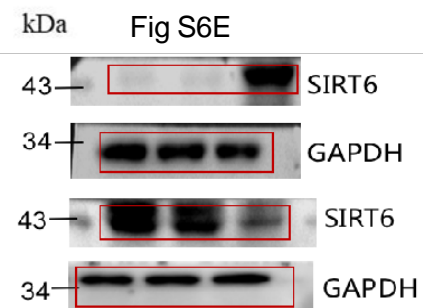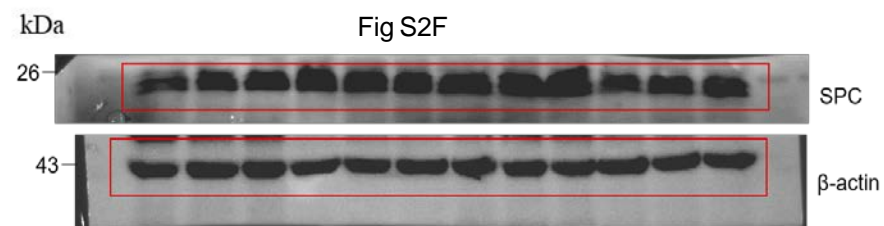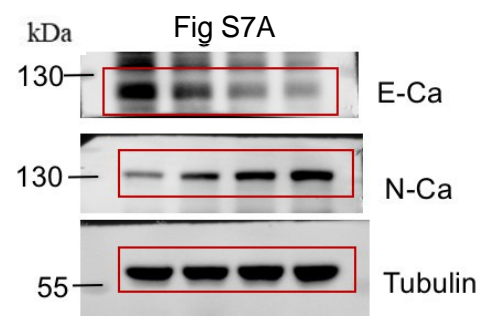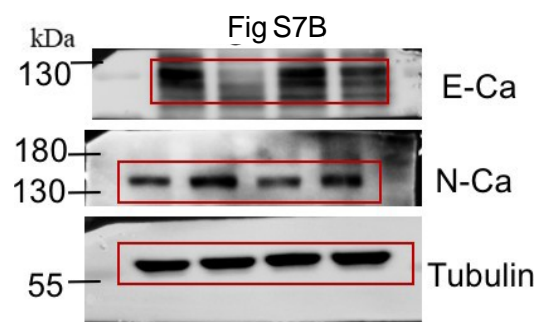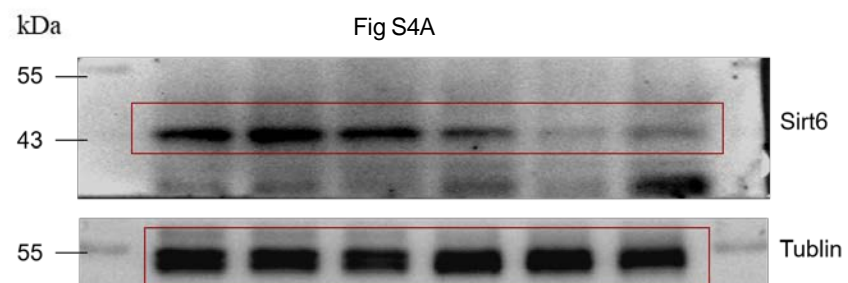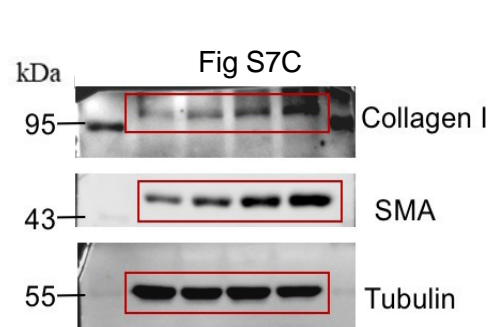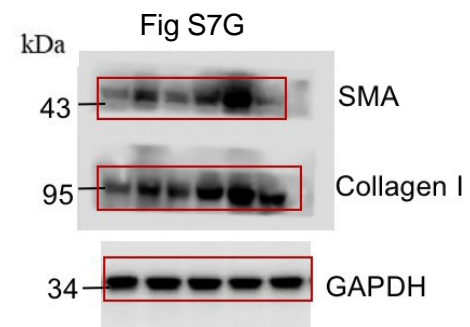

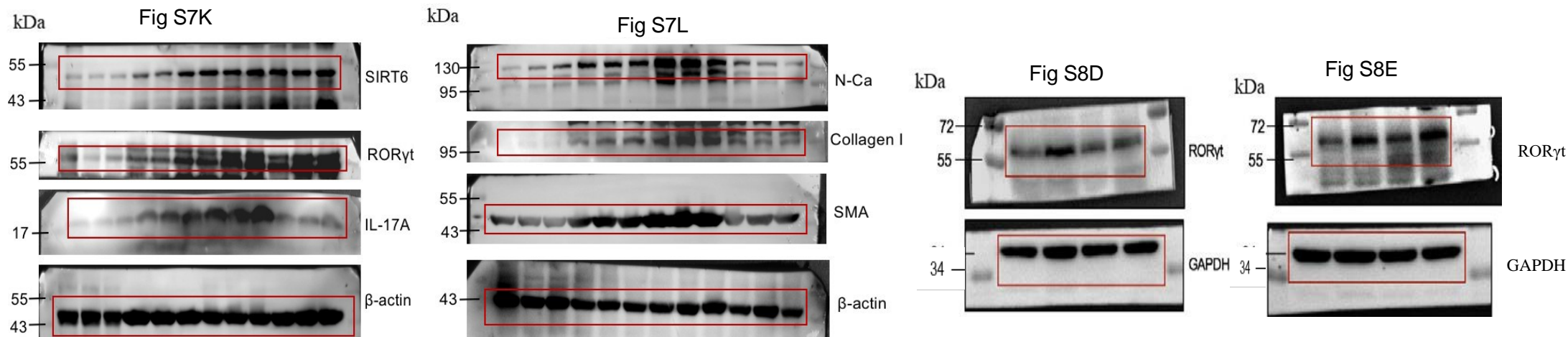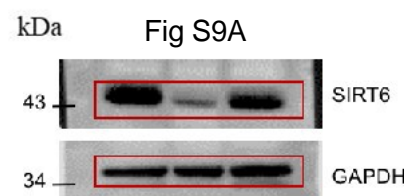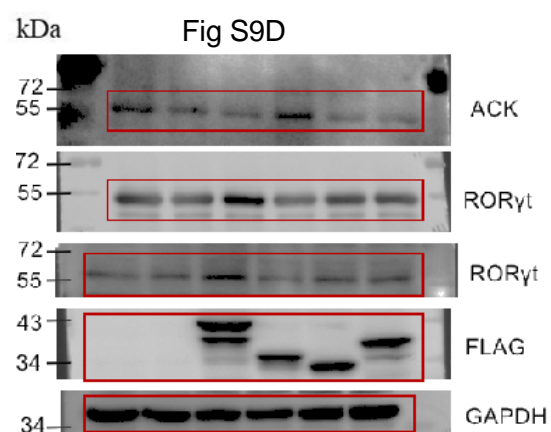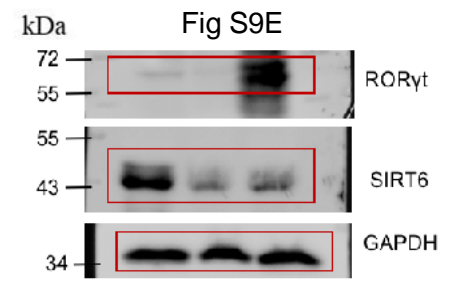

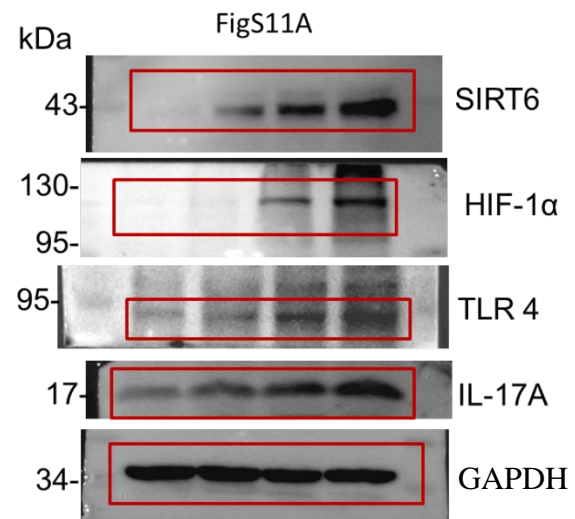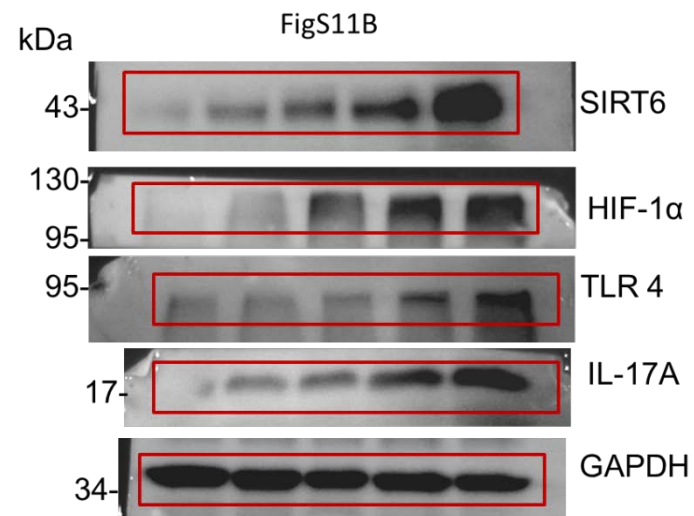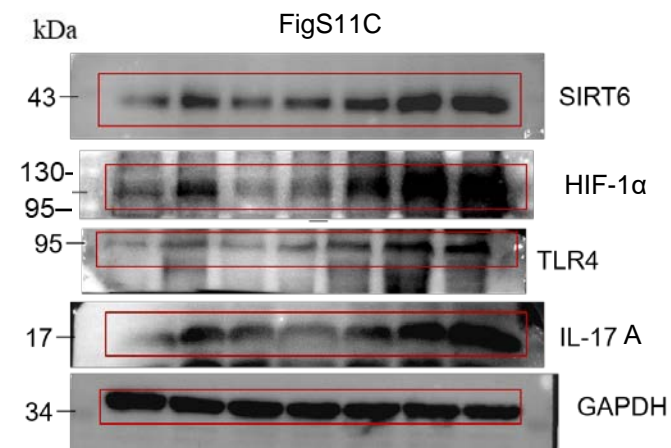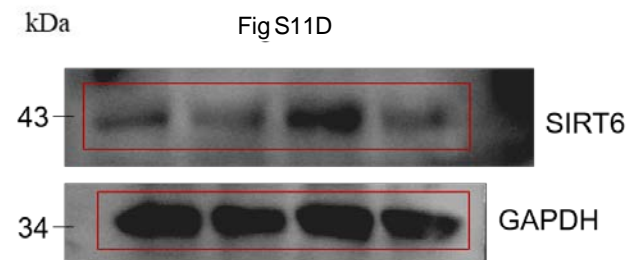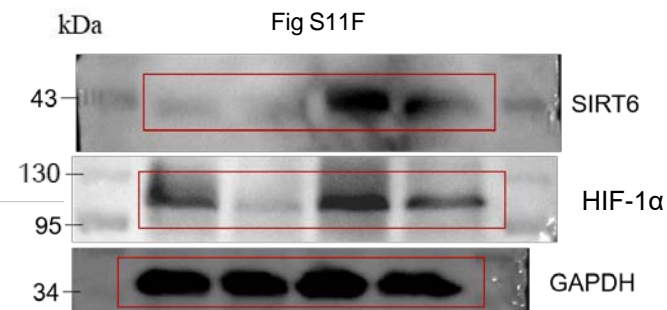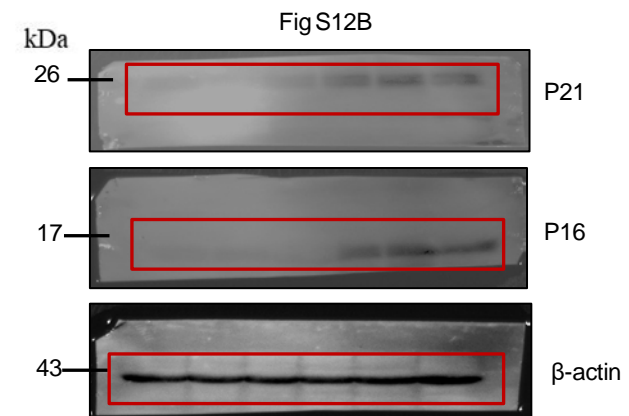

Supplement: Supplementary file 8 — Source Data [file 41467_2023_44179_MOESM8_ESM.zip › Source Data/File 2 Uncropped western blot images.pdf]
